# Exploring Postharvest Metabolic Shifts and NOX2 Inhibitory Potential in Strawberry Fruits and Leaves via Untargeted LC-MS/MS and Chemometric Analysis

**DOI:** 10.3390/metabo15050321

**Published:** 2025-05-13

**Authors:** Georgia Ladika, Paris Christodoulou, Eftichia Kritsi, Thalia Tsiaka, Georgios Sotiroudis, Dionisis Cavouras, Vassilia J. Sinanoglou

**Affiliations:** 1 Laboratory of Chemistry, Analysis & Design of Food Processes, Department of Food Science and Technology, University of West Attica, Agiou Spyridonos, 12243 Egaleo, Greece; gladika@uniwa.gr (G.L.); pchristodoulou@uniwa.gr (P.C.); ekritsi@uniwa.gr (E.K.); tsiakath@uniwa.gr (T.T.); 2 Institute of Chemical Biology, National Hellenic Research Foundation, 48, Vas. Constantinou Ave., 11635 Athens, Greece; gsotir@eie.gr; 3 Department of Biomedical Engineering, University of West Attica, Agiou Spyridonos, 12243 Egaleo, Greece; cavouras@uniwa.gr

**Keywords:** strawberry, postharvest metabolism, LC-MS/MS, chemometric analysis, phenolic compounds, flavonoids, organic acids, plant secondary metabolites, NOX2 enzyme

## Abstract

**Background/Objectives:** Strawberries are highly appreciated for their rich phytochemical composition, but rapid postharvest deterioration limits their shelf life and nutritional quality. This study aimed to investigate the metabolic changes occurring in both strawberry fruits and leaves during storage and to evaluate the NADPH oxidase 2 (NOX2) inhibitory potential of strawberry-derived metabolites. **Methods:** Untargeted LC-MS/MS analysis was conducted on fruit and leaf tissues stored at 8 ± 0.5 °C. A total of 37 metabolites were identified, including organic acids, phenolic acids, flavonoids, and hydroxycinnamic acid derivatives. Multivariate statistical analyses (ANOVA, PLS-DA, and volcano plots) were used to assess temporal and tissue-specific metabolic shifts. Additionally, a machine learning-based predictive model was applied to evaluate the NOX2 inhibitory potential of 24 structurally characterized metabolites. **Results:** Storage induced significant and tissue-specific metabolic changes. In fruits, malic acid, caffeic acid, and quercetin-3-glucuronide showed notable variations, while ellagic acid aglycone and galloylquinic acid emerged as prominent markers in leaves. The predictive model identified 21 out of 24 metabolites as likely NOX2 inhibitors, suggesting potential antioxidant and anti-inflammatory bioactivity. **Conclusions**: These findings provide new insights into postharvest biochemical dynamics in both strawberry fruits and leaves. The results highlight the value of leaves as a source of bioactive compounds and support their potential valorization in functional food and nutraceutical applications.

## 1. Introduction

Strawberries (*Fragaria × ananassa*) are among the most widely consumed fruits globally, appreciated for their vibrant color, distinct aroma, and rich nutritional profile. They are an excellent source of vitamins, organic acids, flavonoids, and phenolic compounds, which contribute to their high antioxidant capacity and associated health benefits [1,2,3]. However, despite their nutritional and economic value, strawberries are highly perishable, exhibiting rapid postharvest deterioration due to their high water content, metabolic activity, and susceptibility to microbial decay [4,5,6]. Understanding the biochemical mechanisms underlying postharvest changes is crucial for developing strategies to enhance fruit quality, extend shelf life, and reduce postharvest losses. Once harvested, strawberries undergo significant biochemical changes, including the degradation of organic acids, enzymatic oxidation of phenolic compounds, and fluctuations in antioxidant metabolites, leading to quality deterioration and reduced storage stability. Among these metabolic changes, organic acid metabolism plays a central role in maintaining fruit acidity, flavor, and overall metabolic balance [7]. Malic and citric acids are the predominant organic acids in strawberries, and their degradation has been linked to postharvest senescence, affecting taste and overall fruit stability [8]. In parallel, phenolic compounds, including flavonoids and phenolic acids, are vital for the fruit’s antioxidant defense and structural integrity [9]. During storage, these compounds undergo significant transformations due to oxidative stress and enzymatic degradation, impacting the fruit’s nutritional value and quality [10,11]. Therefore, strawberries were selected for this study due to their global economic and nutritional significance, as well as their high postharvest perishability. While the fruit is widely consumed and well characterized, the leaves are often discarded despite accumulating evidence of their rich phenolic content and potential health benefits [4].

While much research has focused on postharvest changes in strawberry fruit, the metabolic responses of strawberry leaves during storage remain largely unexplored. Strawberry leaves contain a diverse array of secondary metabolites, including terpenes, flavonoids, and hydroxycinnamic acid derivatives, which are integral to plant defense mechanisms and environmental adaptation [12,13]. However, their metabolic stability or transformations during storage remain poorly understood, raising questions about how their biochemical composition compares to fruit metabolic shifts. Investigating these changes can provide insight into the bioactive potential of strawberry leaves, which are often discarded as waste. Identifying their storage-dependent metabolic profile may open new avenues for their valorization as functional ingredients, aligning with current goals in sustainable food systems and circular agriculture.

Additionally, there is growing interest in identifying dietary antioxidants, that can attenuate enzyme-driven reactive oxygen species (ROS) generation and inflammatory signaling [14]. NADPH oxidase 2 (NOX2) is a major enzymatic source of ROS that drives oxidative stress and persistent inflammation, a key mechanism in chronic diseases such as diabetes and cardiovascular disorders [15]. Notably, strawberry fruit-derived phenolic compounds have demonstrated the capacity to reduce oxidative stress and inflammation, providing a strong rationale for applying machine learning to systematically evaluate strawberry metabolites as potential NOX2 inhibitors [16].

Therefore, this study investigates the postharvest metabolic shifts in strawberry fruits and leaves through LC-MS/MS-based metabolite profiling, providing insights into storage-induced biochemical changes. By examining both fruit and leaf tissues, this study aims to better understand postharvest metabolic changes and explore opportunities for valorizing strawberry by-products. By analyzing fluctuations in key metabolites during storage, the study clarifies their roles in fruit senescence and leaf metabolic adaptation. Advanced multivariate statistical analyses, including Partial Least Squares Discriminant Analysis (PLS-DA) and volcano plot analysis, were employed to identify metabolic markers that differentiate early and late storage stages. This dual focus supports a more holistic and sustainable approach to crop utilization, aligned with current trends in functional foods and plant-based bioactives. Additionally, a machine learning-based evaluation of the NOX2 inhibitory potential of strawberry-derived metabolites was performed to systematically assess their role as potential modulators of oxidative stress pathways. By integrating biochemical profiling, statistical modeling, and predictive machine learning, this research enhances the understanding of postharvest metabolic differentiation between fruits and leaves, with implications for optimizing storage conditions, preserving antioxidant properties, and improving postharvest management strategies.

## 2. Materials and Methods

### 2.1. Strawberry Fruit and Leaf Samples and Storage Conditions

This study was conducted on hydroponically cultivated strawberries (*Fragaria × ananassa* cv. Marisol), sourced from K & K GREEN FARMS, Kyllíni, Greece. The strawberries and their attached leaves were harvested at their commercially mature stage and promptly transported under cold chain conditions to the laboratory within 24 h postharvest. The initial day of storage (day 1) was defined as the day they arrived at the laboratory.

To assess postharvest metabolic changes, the samples were stored under controlled environmental conditions in a refrigerated incubator (POL-EKO Cooled Incubator ST 3, POL-EKO-APARATURA) set at 8.0 ± 0.5 °C, with relative humidity maintained at 60 ± 2%. Storage continued until spoilage rendered the samples unfit for analysis. The fruits were monitored for up to 11 days, while the leaves were analyzed until day 8, as they exhibited visible deterioration beyond this point.

Before storage, the strawberries and leaves were visually inspected, and any damaged, over-ripe, or diseased samples were removed to ensure experimental consistency. Throughout storage, representative samples were collected at five predefined time points (days 1, 4, 6, 8, and 11 for fruit; days 1, 4, 6, and 8 for leaves) to evaluate progressive metabolite changes. At each time point, 12 individual fruits and their corresponding leaves were randomly selected for metabolite extraction and subsequent analysis.

### 2.2. Metabolite Extraction from Strawberry Fruits and Leaves

To analyze postharvest metabolic changes, metabolite extraction was performed on both strawberry fruits and leaves at designated storage time points (days 1, 4, 6, 8, and 11 for fruit; days 1, 4, 6, and 8 for leaves). At each sampling point, 12 unique fruits or leaves were used to create equal number of sample replicates (12 replicates). For each replicate, 1 g of fresh strawberry fruit or leaf tissue was collected from a single whole fruit (not pooled), comprising both external and internal tissue for fruits, to reflect the overall metabolic composition. The same procedure was followed for leaf tissue. Prior to extraction, each sample was finely chopped and homogenized using a mechanical blender to ensure uniform consistency and maximize the release of the compounds. The homogenized tissue was then mixed with 80% aqueous methanol *(v*/*v*) in a 1:5 (*w*/*v*) ratio, ensuring the efficient extraction of both polar and semi-polar metabolites as described by Ladika et al. [17]. The use of 80% aqueous methanol (*v*/*v*) was based on its well-documented efficiency in extracting a wide range of polar and semi-polar metabolites, and it has been widely used due to its ability to disrupt cell walls and solubilize both hydrophilic and moderately lipophilic compounds, ensuring broad metabolite coverage [18,19]. The mixture was vortexed for 1 min and subsequently incubated at 20 °C for 24 h in sealed containers, allowing for optimal metabolite solubilization. After incubation, the extracts were filtered under vacuum using a Buchner funnel to remove solid residues. The resulting filtrate was then centrifuged at 10,000 rpm for 10 min at 4 °C to further clarify the extract. The supernatant was carefully collected and adjusted to a final volume of 10 mL with 80% aqueous methanol to ensure standardization across all samples. The extracts were then stored at −80 °C until LC-MS/MS analysis to prevent potential degradation. For LC-MS/MS analysis, 1 mL of each extract was acquired and then, the solvent was evaporated to obtain the dry residue containing the metabolites of interest. The extract residues were then reconstituted in 1 mL of LC-MS-grade methanol containing 0.1% *v*/*v* formic acid. In preparation for analysis, all the reconstituted samples were filtered using Chromafil Xtra PET 0.45 μm syringe filters (Macherey-Nagel, Düren, Germany).

### 2.3. LC-MS/MS Analysis

The extracted metabolites from strawberry fruits and leaves were analyzed using Liquid Chromatography–Electrospray Ionization Tandem Mass Spectrometry (LC-ESI(-)-MS/MS) to investigate postharvest metabolic alterations. The chromatographic system consisted of an Agilent 1200 HPLC (Agilent Technologies, Santa Clara, CA, USA) coupled to a 3200 Q TRAP triple-quadrupole linear ion trap mass spectrometer (Sciex, Framingham, MA, USA) equipped with an ESI source operating in negative ionization mode, which is favorable for the analysis of phenolic compounds. Separation was achieved on an Eclipse Plus C18 reversed-phase column (50 mm × 2.1 mm i.d., 3.5 µm particle size) with an in-line filter (2.1 mm, 0.2 µm). The mobile phase consisted of water with 0.2% (*v*/*v*) formic acid (Solvent A) and acetonitrile with 0.1% (*v*/*v*) formic acid (Solvent B), applied in a binary gradient.

Mass spectrometric data were acquired in information-dependent acquisition (IDA) mode using enhanced product ion (EPI) scans for MS/MS fragmentation. The mass accuracy parameters were set at 0.1 Da for MS and 0.5 Da for the MS/MS spectra. All instrument settings followed previously validated protocols developed by our research group [20,21]. Data analysis was performed using Analyst 1.6 software, and metabolite semi-quantitative comparisons were based on relative peak intensities. Each sample was analyzed in triplicate. All samples were subjected to analysis in a random order.

For metabolite identification, the MS/MS fragmentation spectra were compared with previously reported data from published studies on strawberry and other plant-derived metabolites [12,22,23,24,25,26,27,28,29,30,31]. Tentative identification was achieved when at least two characteristic fragment ions and the parent (precursor) ion matched the fragmentation patterns described in the literature, ensuring higher confidence in the annotation of the detected compounds.

### 2.4. Statistical Analysis

The metabolic dataset obtained from LC-MS/MS analysis was subjected to statistical analysis to assess postharvest metabolic fluctuations in strawberry fruits and leaves. All statistical analyses were performed using the MetaboAnalyst 6.0 platform. To determine significant differences in metabolite intensities across storage days, one-way analysis of variance (ANOVA) was performed, followed by Tukey’s Honest Significant Difference (HSD) post hoc test. A significance threshold of *p <* 0.05 was used to identify metabolites with statistically significant differences over time.

Additionally, volcano plot analysis was employed using a Wilcoxon non-parametric test, considering metabolites with *p* < 0.05 and a fold change greater than 1.0 as significant. In these plots, the x-axis represents the log2 fold change (log2(FC)) to indicate the magnitude and direction of metabolite variation, while the y-axis displays the −log10 (*p*-value) to highlight statistical significance.

For multivariate analysis, data normalization was applied, including autoscaling, to ensure comparability between metabolites with different concentration ranges. Supervised Partial Least Squares Discriminant Analysis (PLS-DA) was then applied to assess whether metabolite fluctuations could differentiate storage time points. The quality of the PLS-DA models was evaluated based on the goodness-of-fit parameter (R^2^) and predictive ability (Q^2^). The reliability of the classification models was further validated using permutation tests (n = 1000).

### 2.5. Machine Learning-Based Evaluation of Strawberry Metabolites for NOX2 Inhibitory Potential

#### 2.5.1. Curation of NOX2 Inhibitor Dataset and Molecular Descriptors Calculation

Testing compounds against NADPH oxidase 2 (NOX2) were retrieved from the ChEMBL database (https://www.ebi.ac.uk/chembl/) (accessed on 8 February 2025), ensuring only high-quality entries with verified biological activity were included. Specifically, a dataset of 179 compounds including 107 actives (30 nM < IC_50_ < 99 uM) and 72 not-active compounds (IC_50_ > 100 uM) was created. The molecular descriptors of the dataset were calculated using RDKit (Release_2025.03.1), an open-source cheminformatics software (https://www.rdkit.org/) (accessed on 8 February 2025), to characterize the chemical and physicochemical properties relevant to bioactivity prediction.

Feature (i.e., descriptor) reduction was used to lessen the number of descriptors to a subset of high-discrimination descriptors before being used to design the machine learning system (ML-system). The descriptors’ importances were evaluated using the model.feature_importance_ function provided by the scikit-learn library, where “model” is the used machine learning classifier. We employed four (4) classifiers (Random Forest, CART, XGBoost, and ExtraTrees) to evaluate the importance of the descriptors. Next, descriptors were ranked according to their discriminatory importance. Descriptors’ frequencies of occurrence, at highly ranked positions of importance, were recorded using the 4 classifiers over 100 repetitions to evaluate the above-named function, i.e., 400 descriptor-importance evaluation cycles in total. The highest ranked 16 descriptors that consistently exhibited high importance in all evaluation cycles were used in the design of the ML-system.

#### 2.5.2. Machine Learning Analysis

Each of the four classifiers (Random Forest, CART, XGBoost, and ExtraTrees) was separately trained on the retrieved ChEMBL dataset and the 16 highly important descriptors. Thus, the dataset consisted of two classes, the active class of 107 active molecules of 16 descriptors and the not-active class of 72 not-active molecules of 16 descriptors.

The best ML-system was designed using the following procedure. First, the dataset was randomly split into two parts; the first contained 70% of the data, called the training dataset, and it was used for the design of the ML-system, and the second, called the testing dataset, comprised 30% of the data, and was used for evaluating the performance of the designed ML-system on unseen dataset, i.e., how well the designed ML-system would perform when presented at its input with a new dataset for classification. Second, the data of the training dataset was normalized (Equation (1)) by subtracting from each descriptor value its mean value (over both classes) and dividing this by the corresponding standard deviation (again over both classes).

normalized value = (descriptor value-mean)/standard deviation(1)

Third, both classes (active and not-active) of the training dataset were equalized in terms of numbers of members using the SMOTE function of the scikit-learn library for classifier performance reasons. Fourth, for a particular classifier and an increasing number of the 16 high-importance descriptors (i.e., the first 2, 3, …,16 features), the ML-system was designed, and its performance at each descriptor combination was evaluated using the repeated KFOLD method from the scikit-learn library. The highest-performing ML-system design for the particular classifier and the best descriptor combination was used to classify the left-out testing dataset. Before being presented at the input of the best ML-system, the testing dataset underwent normalization similar to that of the training dataset; however, this used the mean value and the standard of the training dataset. Fifth, the whole process of the 4 previous steps was repeated 5 times, and the classification accuracy, active-class accuracy, not-active-class accuracy, and the area under the curve (AUC) of the Receiver Operating Characteristic curve (ROC_AUC) were recorded. Finally, the whole process of the 5 design steps was repeated for each one of the 4 classifiers, and it was thus possible to identify the best-performing design, i.e., the combination of the classifier (XGBoost) and the number of features (the first 11 features of high importance) employed in the best ML-system design.

#### 2.5.3. Machine Learning System Deployment to Predict Bioactivity of Identified Strawberry Metabolites by LC-MS/MS Analysis

Following the design of the best ML-system, the latter was redesigned, but this time, the total of the dataset from the ChEMBL library (170 compounds, 107 active and 72 not-active) was employed to construct the ML-system using the best-performing classifier and descriptors. The ML-system was next saved on a disk together with the means and standard deviations of the employed descriptors. The bioactivity of the metabolites, identified by the LC-MS/MS analysis of strawberry fruits and leaves, was then checked by the ML-system. Specifically, 24 out of the 37 metabolites were selected, since they were described by an accurate molecular structure. Their SMILES structures were retrieved and their descriptors were calculated using the RDKit python library. Next, the values of the 11 best descriptors were normalized using the saved means and standard deviations of the designed ML-system. The 24 metabolites and the 11 descriptors were presented at the input of the ML-system. The latter were read from disk and used to classify the 24 LC-MS/MS-identified metabolites into active or not active. The classification was recorded.

The above process was repeated for each of the 3 remaining classifiers included in the present study (Random Forest, CART, ExtraTrees). Metabolites classified into the same class by all 4 classifiers were accepted as of true bioactivity indication.

**Figure 1:**
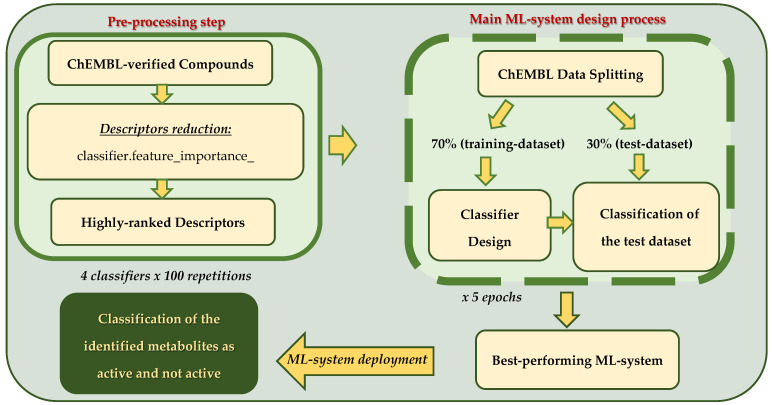
Flowchart of machine learning procedure.

The following figure (Figure 1) is a representative flowchart of the above-mentioned machine learning procedure.

## 3. Results

### 3.1. Metabolite Profile of Strawberry Fruits and Leaves

The application of LC-MS/MS analysis led to the identification of 37 metabolites in the strawberries’ fruits and leaves belonging to various biochemical classes, including organic acids, phenolic acids, flavonoids, hydroxycinnamic acid derivatives, amino acids, terpenoids, and fatty acid glycosides. The metabolite distribution varied distinctly between the fruits and leaves, reflecting differential tissue-specific metabolic pathways and physiological functions. Among these, 25 metabolites were found in the fruit, 22 were found in the leaves, and 10 were found in both. The presence of common compounds suggests coordinated metabolic pathways or potential metabolite transfer between the fruit and leaves, while exclusive compounds highlight tissue specialization. A comprehensive overview of these metabolites, including their molecular weight (MW), negative [M−H]^−^ ions, MS/MS fragmentation information, retention time, and tissue distribution, is presented in Table 1. The identification of phenolic compounds was achieved through the comparative analysis of the parent ions (*m*/*z*) obtained from the MS spectra and the fragments from the MS/MS spectra with corresponding literature data.

Among the organic acids, malic and citric acids were detected in both strawberry fruits and leaves, while L-ascorbic and dehydroascorbic acids were found exclusively in the fruits. Phenolic acids such as caffeic acid, ellagic acid aglycone, and caffeic acid hexoside were present in both tissues. Gallic acid monohydrate, coumaric acid hexose, and dicaffeoylquinic acid were specific to strawberry fruits, while p-coumaroyl ester, galloyl hexose, coumaroylquinic acid, and galloylquinic acid were exclusive to leaves. Flavonoids were found to be distributed widely in both tissues. Epicatechin, quercetin-3-glucuronide, flavan-3-ol derivatives, kaempferol coumaroyl hexose, phloridzin, and kaempferol rutinoside were detected in both strawberry fruits and leaves. Conversely, apigenin-7-O-glucoside, kaempferol glucuronide, kaempferol acetylglucoside, and quercetin hexoside were exclusive to the fruits, while kaempferol hexose, kaempferol pentose glucuronide, and quercetin rutinoside were detected only in the leaves. Hydroxycinnamic acid derivatives were identified in both tissues. Ferulic acid hexose derivative was found only in strawberries’ fruits, while dihydroferulic acid 4-O-glucuronide and di-coumaroyl hexose were found exclusively in the leaves. Ellagic acid deoxyhexose was present in both tissues. Salidroside, a phenolic glycoside with known bioactivity, was detected only in the leaves. Distinct separation was also seen in terpenoids and fatty acid derivatives. Sesquiterpenoids and sapogenins were fruit-specific, while octadecatrienoic acid glycoside was exclusive to leaves. Tryptophan, the only identified amino acid, was detected only in the fruits.

### 3.2. Statistical Analysis of Metabolite Changes During Storage in Strawberry Fruits

#### 3.2.1. ANOVA Post Hoc Analysis of Metabolite Fluctuations in Strawberry Fruits

A one-way analysis of variance (ANOVA) with Tukey’s HSD post hoc test was conducted to assess the significant fluctuations in metabolite intensities over different days of storage in strawberries. The analysis revealed seven metabolites that exhibited statistically significant changes (*p <* 0.05), indicating metabolic shifts during the postharvest storage period (11 days). Among the organic acids, malic acid demonstrated a steady decrease over the 11-day storage period, while citric acid exhibited an initial increase before stabilizing. Phenolic acids displayed distinct trends, with caffeic acid showing a continuous decrease, suggesting progressive metabolic transformation or degradation, while ferulic acid hexose derivative and dicaffeoylquinic acid exhibited fluctuations during storage. Kaempferol glucuronide also varied significantly over time, suggesting changes in flavonoid metabolism during storage. The statistical significance of these fluctuations is summarized in Table 2, while box plots illustrating the key metabolites’ trends are illustrated in Figure S1 (Supplementary Materials).

#### 3.2.2. Discriminant Analysis of Metabolite Variability in Strawberry Fruits During Storage

**Figure 2:**
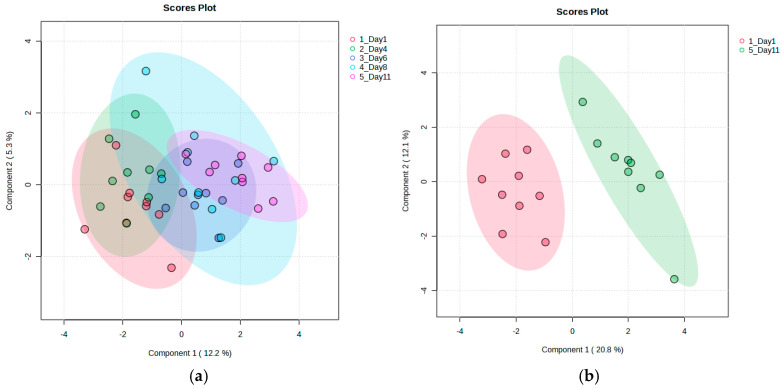
Partial Least Squares Discriminant Analysis (PLS-DA) scatter plots showing metabolic differentiation in strawberry fruits during storage: (**a**) a comparison across all storage days and (**b**) a focused comparison between day 1 and day 11. While Component 1 and Component 2 individually explain a relatively small percentage of the total variance—an expected outcome in complex datasets—together they capture a meaningful separation between time points. The model focusing on early- and late-stage changes (**b**) was validated through permutation testing, confirming its reliability in identifying storage-related metabolic shifts.

To discriminate between storage days, Partial Least Squares Discriminant Analysis (PLS-DA) was conducted. When considering all storage days, no strong classification accuracy was observed. However, a subtle trend of metabolic changes from day 1 to day 11 was observed, as presented in Figure 2a. This finding suggests that postharvest metabolic variability may occur progressively rather than in sharply defined phases. A focused comparison of the first (day 1) and last (day 11) storage days resulted in a substantial improvement in classification accuracy (0.95), confirming that metabolic changes led to a distinct differentiation between the early and late storage stages. The scatter plots of the discriminant analysis for all storage days, as well as the focused comparison of day 1 and day 11, are shown in Figure 2. The validity and robustness of the PLS-DA model designed to discriminate between the first and last day of storage was confirmed through a permutation test, and the corresponding permutation plot, as well as a table summarizing the model parameters (R^2^, Q^2^, and the two-component accuracy), can be found in Figure S2 in the Supplementary Materials.

**Figure 3:**
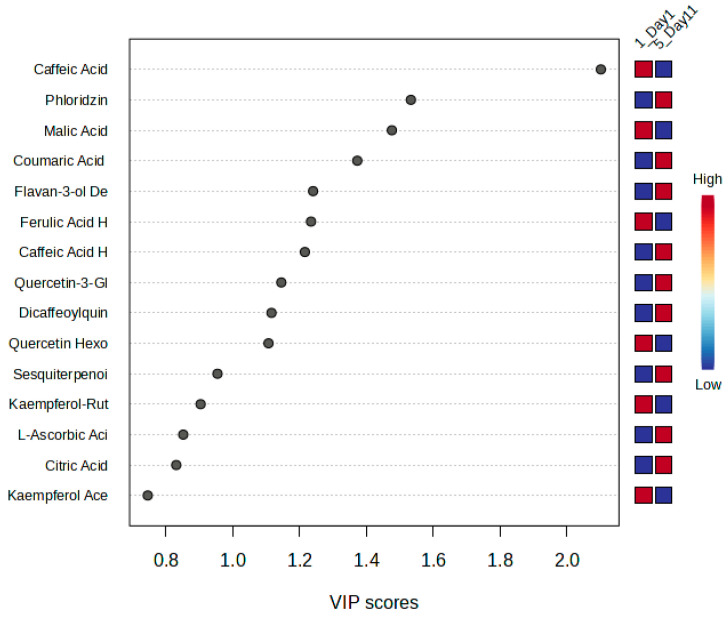
VIP scores of metabolites contributing to storage-related metabolic discrimination (day 1 vs. day 11).

The variable importance in projection (VIP) plot identified the key metabolites responsible for differentiating between day 1 and day 11. Caffeic acid exhibited the highest VIP score, highlighting its significant depletion over storage and reinforcing its role as a major antioxidant compound. Malic acid was also identified as a significant metabolite, reflecting its steady consumption as a respiratory substrate throughout the storage period. Phloridzin, a dihydrochalcone glycoside, emerged as another key contributor, exhibiting fluctuations potentially linked to sugar metabolism and structural defense. Its involvement in regulating cell wall integrity and reactive oxygen species has been demonstrated in other plant systems, where it plays a crucial role in development and pathogen resistance [33]. Additional contributors included coumaric acid, flavan-3-ol derivatives, ferulic acid hexose derivative, caffeic acid hexoside, and quercetin-3-glucuronide, all of which are involved in antioxidant defense, flavonoid metabolism, and structural modifications [34,35,36,37]. The ranking of these metabolites based on their VIP scores is shown in Figure 3.

**Figure 4:**
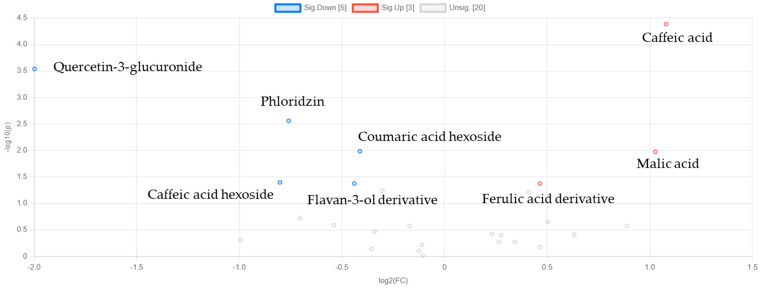
Volcano plot showing key upregulated and downregulated metabolites in strawberries. The numbers in square brackets in the legend indicate the number of significantly upregulated, and downregulated metabolites, respectively.

The volcano plot of the day 1 vs. day 11 comparison of strawberries revealed key metabolites contributing to the metabolic differentiation between the early and late storage stages (Figure 4 and Table S1 in the Supplementary Materials). Several metabolites demonstrated significant and substantial alterations. Caffeic and malic acid were among the most reduced on day 11, as indicated by their positive log2 (FC) values (1.08 and 1.03, respectively) and significant *p*-values (<0.001 and <0.05, respectively), suggesting their depletion during storage. In contrast, quercetin-3-glucuronide exhibited the most pronounced increase, with a log2 (FC) of −1.99 and a highly significant *p*-value (<0.001), pointing to its marked accumulation. Phloridzin, coumaric acid hexose, flavan-3-ol derivative, and caffeic acid hexoside also increased significantly (*p <* 0.05) during storage, consistent with a progressive alteration of flavonoid and phenolic acid metabolism. Finally, ferulic acid hexose derivative exhibited a significant (*p <* 0.05) decrease.

### 3.3. Statistical Analysis of Metabolite Changes During Storage in Strawberry Leaves

#### 3.3.1. ANOVA Post Hoc Analysis of Metabolic Alterations in Strawberry Leaves

A one-way analysis of variance with Tukey’s HSD post hoc test was conducted to examine whether any metabolic changes occurred in strawberry leaves across different storage days. Nine metabolites displayed statistically significant changes (*p <* 0.05), thus indicating metabolic alterations in response to postharvest storage conditions. The statistical significance of these alterations is outlined in Table 3, and a graphical representation of the key metabolites’ trends is presented in Figure S3 (Supplementary Materials).

Among the organic acids, malic acid levels exhibited temporal alterations throughout storage, while citric acid levels initially increased on day 4 and subsequently decreased on later storage days. Salidroside, identified as a phenolic glycoside, also exhibited significant variations, with a notable rise on day 4 followed by a gradual decline toward the end of storage. Ellagic acid aglycone increased progressively to day 8, indicating a potential role in stress adaptation. Hydroxycinnamic acid derivatives, such as galloylquinic acid and galloyl hexose, demonstrated dynamic shifts, with galloylquinic acid reaching a marked peak on day 4. Flavonoid-related metabolites, including caffeic acid hexoside and flavan-3-ol derivative, fluctuated during storage, ultimately showing a decreasing trend by the end of the storage period.

#### 3.3.2. Discriminant Analysis of Metabolite Variability in Strawberry Leaves During Storage

**Figure 5:**
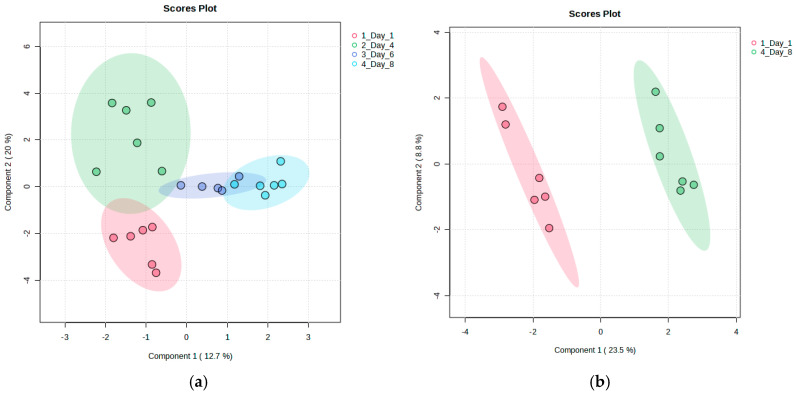
PLS-DA scatter plots illustrating the metabolic changes in strawberry leaves during storage: (**a**) across all sampling days; (**b**) a comparison between day 1 and day 8. The low variance explained by Component 1 and Component 2 reflects the high dimensionality and subtle variability inherent in metabolomics data. Despite this, the model summarizing the metabolic alterations between early and late storage stages (**b**) exhibited good classification accuracy and passed permutation testing, supporting its robustness in distinguishing between early and late storage stages.

Partial Least Squares Discriminant Analysis (PLS-DA) was performed over all the time points. The model achieved a classification accuracy of 0.78, with a permutation test yielding *p* = 0.04, thus confirming the robustness of the discriminant analysis. Subsequently, discriminant analysis was performed, focusing on the initial (day 1) and final (day 8) storage days, with the objective of comprehensively understanding the metabolic alterations between the early and late stages of storage. The scatter plots for both discriminations are presented in Figure 5. The validity and robustness of the PLS-DA model designed to discriminate between the first and last day of storage (Figure 2b), along with a table summarizing the model parameters, can be found in Figure S4 of the Supplementary Materials.

**Figure 6:**
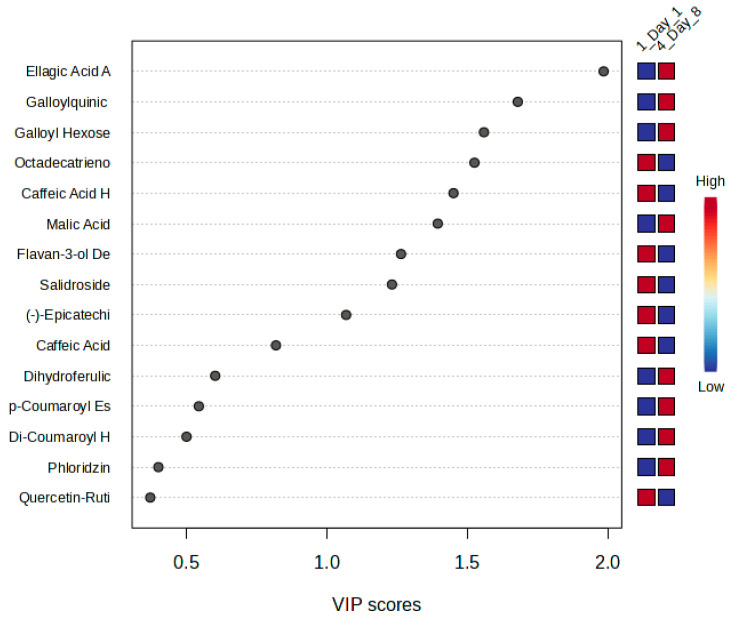
VIP scores of metabolites contributing to storage-related metabolic discrimination (day 1 vs. day 8).

The VIP plot identified the most important metabolites driving the metabolic changes between day 1 and day 8 (Figure 6). The metabolites that most significantly contributed to the discrimination included ellagic acid aglycone, which exhibited the strongest contribution, followed by galloylquinic acid, galloyl hexose, octadecatrienoic, caffeic acid hexoside, malic acid, flavan-3-ol derivative, salidroside, and epicatechin. These metabolites played a significant role in distinguishing the metabolic state of the leaves at the beginning and end of storage.

**Figure 7:**
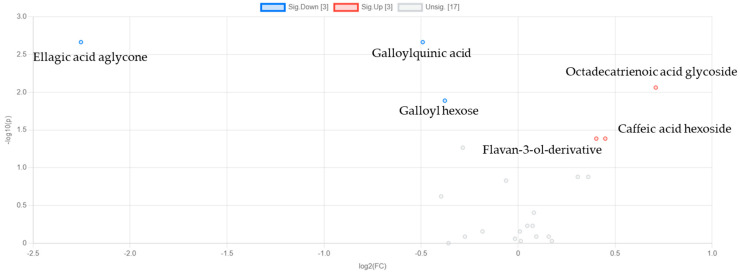
Volcano plot showing key upregulated and downregulated metabolites. The numbers in square brackets in the legend indicate the number of significantly upregulated, and downregulated metabolites, respectively.

To further explore the key contributors and the magnitude of the metabolic changes during storage, a volcano plot analysis was performed for day 1 vs. day 8 in strawberry leaves (Figure 7). The results of the analysis indicated that ellagic acid aglycone underwent the most significant increase, with a log2 (FC) of −2.25 and a *p*-value of 0.002, suggesting a pronounced accumulation during storage. Galloylquinic acid and galloyl hexose also showed significant (*p <* 0.05) increases, while octadecatrienoic acid glycoside, caffeic acid hexoside, and flavan-3-ol derivative showed significant (*p <* 0.05) reductions. These findings reveal that both phenolic acids and flavonoid-related compounds underwent marked fluctuations. The exact fold changes and *p*-values for the significantly altered metabolites are provided in Table S2 (Supplementary Materials).

#### 3.3.3. Machine Learning-Based Evaluation of NOX2 Inhibitory Potential

Four classifiers (Random Forest, CART, XGBoost, and ExtraTrees) were employed to rank the molecular descriptors concerning their discriminatory importance. Sixteen (16) descriptors were found to be of high importance regarding NOX2 inhibitory activity for the CHEMBL database compounds. The selected descriptors, presented in Table 4, represent essential physicochemical properties of molecules that demonstrate their importance for bioactivity.

The BCUT2D descriptors, including BCUT2D_MWHI, BCUT2D_MRHI, and BCUT2D_MWLOW, emerge as the top features since they function as eigenvalue metrics from weighted molecular adjacency matrices. BCUT2D indices demonstrate how molecular size and shape together with atomic weight distribution reveal that molecular bulk and polarizability determine NOX2 inhibitory potential. The top-ranking descriptors include several Van der Waals surface-area-based features such as PEOE_VSA6/9 and SMR_VSA10 along with SlogP_VSA6 and VSA_EState2/6/8. The descriptors categorize molecular surface areas by partial charge (PEOE), polarizability (SMR), and lipophilicity (SlogP), which indicates that balancing polar and hydrophobic regions on the surface is crucial for activity. The surface area descriptors PEOE_VSA6 and PEOE_VSA9 illustrate that an ideal polar surface interaction with the NOX2 enzyme is essential. Also, VSA_EState descriptors indicate that certain electron-rich or electron-deficient surface areas are associated with inhibitory functionality. The valence and simple connectivity indices of order 2 (Chi2v and Chi2n) are presented with high importance because they connect molecular topology and branching patterns to biological activity, such as aromatic ring presence or substituent complexity. The sp^3^ carbon fraction (FractionCSP3) serves as a critical feature because it displays that enzyme activity depends on the level of saturation which differentiates between aliphatic and aromatic content, as structures with high aromaticity (low sp^3^ fractions) exhibit different interaction capabilities compared to aliphatic structures. The presence of the quantitative estimate of drug-likeness (QED) among leading features denotes that tested NOX2 inhibitors manifest a balanced combination of physicochemical properties (such as molecular weight and lipophilicity). The significance of the MinAbsEStateIndex (minimum absolute electrotopological index) reveals how extreme electronic environments including powerful electron-withdrawing/donating substituents and symmetrical charge distributions influence activity [38].

**Figure 8:**
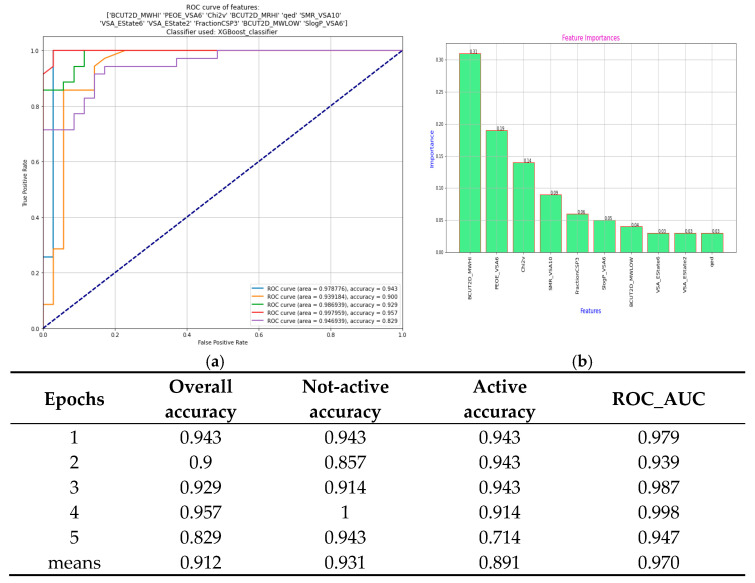
XGBoost classifier 5-epoch results. (**a**) 5-epoch ROC-curves; (**b**) importance of descriptors in classification.

Using these 16 key descriptors, a total of four ML-systems based on four different classifiers (Random Forest, CART, XGBoost, and ExtraTrees) were designed. The best performance was achieved by the XGBoost-based ML-system, and a combination of the first 11 descriptors (Table 4) was utilized for the model generation. The variability of the specific descriptors along the two categories is illustrated in Figure S5 (Supplementary Materials). The XGBoost classifier performed with five epochs resulted in a mean accuracy of 91.2% and demonstrated balanced performance in predicting active and not-active compounds with 89.1% sensitivity for active and 93.1% specificity for not-active compounds. Figure 8a presents the Receiver Operating Characteristic (ROC) curves for five independent epochs of training/testing. The area under each curve (AUC) quantifies the classification performance for NOX2 inhibitor prediction. AUC values close to 1.0 indicate excellent model accuracy, with the mean AUC reaching 0.970, reflecting high sensitivity and specificity. In parallel, Figure 8b shows the descriptors’ importance ranking, identifying the relative contribution of the top molecular descriptors used by the XGBoost classifier. These features, such as BCUT2D_MWHI and PEOE_VSA6, were identified as the most influential in distinguishing active from inactive compounds, based on their role in minimizing classification error. The table represents the five-epoch overall, not-active, and active accuracy, as well as the area under the curve value.

The designed model was then utilized to predict NOX2 inhibitory potential in 24 metabolites derived from strawberries identified via LC-MS/MS analysis. These metabolites represent various chemical classes such as phenolic acids, flavonoids, tannins, terpenoids, and organic acids.

Considering only metabolites classified into the same class by all four of the ML-systems, 21 out of the 24 (88%) tested metabolites were predicted to be potential NOX2 inhibitors with high confidence. The classification of 21 compounds as “Active” highlights the potential of structurally diverse strawberry phytochemicals as potential inhibitors of the NADPH oxidase (NOX2) enzyme complex. The full list of the tested metabolites, along with their corresponding SMILES structures, and the ML prediction is provided in Table 5.

## 4. Discussion

This study provides a comprehensive investigation into the postharvest metabolic dynamics of strawberry fruits and leaves using LC-MS/MS characterization combined with multivariate statistical and cheminformatics tools. Postharvest quality loss and the underutilization of plant by-products remain major challenges in horticulture and food systems. By profiling metabolite alterations during storage, we gain valuable insights into the biochemical shifts that underpin fruit senescence and leaf degradation. These findings not only contribute to our understanding of strawberry postharvest physiology but also highlight the untapped potential of strawberry leaves as reservoirs of bioactive compounds. The integration of the identified metabolites with statistical modeling and bioactivity prediction enables a multidimensional evaluation of plant material, supporting its more sustainable use and value-added applications in both food- and health-related industries.

### 4.1. Metabolic Changes in Strawberry Fruits During Storage

The observed metabolic alterations in strawberries during storage highlight key biochemical processes affecting organic acid, phenolic acid, and flavonoid metabolism. The significant decrease in malic acid suggests its active participation as a respiratory substrate in the TCA cycle, supporting previous studies reporting the involvement of organic acids in sustaining energy demands in stored strawberries [39]. The initial increase in citric acid, followed by stabilization, may be attributed to the conversion of malic acid to citric acid via oxaloacetic acid, a mechanism previously described in strawberries under storage conditions [40]. This suggests that, rather than being rapidly degraded, organic acids are initially transformed into other metabolites, leading to their gradual decrease throughout the storage period. However, the overall decrease in both malic and citric acids in this study confirms that organic acids are progressively exhausted during storage, supporting findings that their depletion contributes to postharvest metabolic adjustments in strawberries [41,42]. Regarding phenolic acid metabolism, a significant decrease in caffeic acid and ferulic acid hexose derivative was observed, suggesting active phenolic degradation during storage, as has been reported by various studies [41,43]. The decreasing trend of ferulic acid hexose aligns with previous reports stating that ferulic acid derivatives reduce due to oxidative stress and enzymatic degradation, leading to reduced antioxidant capacity in stored strawberries over time [44]. Conversely, dicaffeoylquinic acid exhibited an increasing trend over storage, suggesting its role as a secondary metabolite accumulating in response to postharvest conditions. This finding is consistent with the observations made in over-ripe strawberries, where dicaffeoylquinic acid accumulation has been linked to enzymatic transformation during senescence [45]. Its accumulation may be driven by oxidative-stress-related enzymatic activity, contributing to phenolic metabolism during storage. A similar trend was observed in blackthorn (*Prunus spinosa* L.) fruits, where dicaffeoylquinic acids were identified in over-ripe samples, indicating their association with fruit maturation and senescence processes [46]. Flavonoid metabolism followed a mixed trend. While most flavonoids degraded over time, kaempferol acetylglucoside remained relatively stable, aligning with studies showing that some flavonoids accumulate under specific storage conditions [45,47,48]. The variations observed in kaempferol derivatives suggest that, while oxidative stress depletes some flavonoids, others may be upregulated in response to storage conditions.

Multivariate statistical analysis using PLS-DA revealed that metabolic changes in strawberries possibly occurred progressively throughout storage, with a clear discrimination between days 1 and 11, suggesting that specific metabolic markers characterize postharvest biochemical differentiation. VIP scores highlighted caffeic acid, phloridzin, malic acid, and coumaric acid hexoside as the most important contributors, emphasizing their involvement in organic acid metabolism, phenolic degradation, and flavonoid stability during storage. These findings align with previous studies identifying organic acids and flavonoids as key metabolic markers of storage-related transformations [47,49]. Notably, phloridzin exhibited significant variations, which could reflect its sensitivity to oxidative and metabolic changes during storage. This aligns with its known role as a flavonoid involved in stress responses in plant tissues [50]. The volcano plot analysis further pinpointed caffeic acid, quercetin-3-glucuronide, phloridzin, coumaric acid hexose, malic acid, caffeic acid hexoside, flavan-3-ol derivative, and ferulic acid hexose derivative as significantly altered metabolites. The reduction in ferulic acid hexose derivative suggests that oxidative degradation pathways contribute to postharvest quality loss, as research indicates that postharvest decay in strawberries can result from physiological factors, including oxidative stress, leading to the degradation of phenolic compounds like ferulic acid derivatives [51]. Meanwhile, the presence of quercetin-3-glucuronide and flavan-3-ol derivatives as significantly altered metabolites suggests that flavonoids undergo structural modifications during storage, potentially as part of oxidative stress responses. Taken together, the PLS-DA and volcano plot analysis consistently revealed that caffeic acid, malic acid, phloridzin, and quercetin-3-glucuronide are critical markers of postharvest metabolic differentiation, highlighting the key roles of phenolic acid metabolism, flavonoid transformations, and organic acid utilization during strawberry storage.

### 4.2. Metabolic Changes in Strawberry Leaves During Storage

Unlike strawberry fruits, strawberry leaves exhibited a different metabolic response to storage, particularly in terms of organic acids and phenolic compounds metabolism. While malic acid decreased in the fruit, its levels in leaves fluctuated over time, suggesting that organic acid metabolism in the leaves is less focused on energy production and more linked to metabolic buffering or stress adaptation. This trend aligns with findings that malic acid in leaves plays diverse roles beyond respiration, including stress tolerance, osmotic regulation, and antioxidant defense [52,53,54]. Flavonoid-related compounds, including caffeic acid hexoside and flavan-3-ol derivative, underwent moderate fluctuations but ultimately decreased by the end of storage. Similarly, in blueberry leaves, flavonoid content was shown to be highly dependent on environmental conditions, with certain compounds persisting under stress-related conditions [55].

The discriminant analysis (PLS-DA) effectively distinguished metabolic changes in strawberry leaves during storage. This result suggests that storage duration significantly influences the leaf metabolite profile, even though some metabolites exhibit variations rather than linear degradation. The discrimination between day 1 and day 8 highlights a systematic progression of metabolic changes over the storage period. The VIP scores identified ellagic acid aglycone, galloylquinic acid, galloyl hexose, octadecatrienoic acid glycoside, caffeic acid hexoside, malic acid, flavan-3-ol derivative, and salidroside as the most important metabolites contributing to classification. An interesting finding was the detection of salidroside, a phenolic molecule characterized by low toxicity and protective effects on the neurological, cardiovascular, hepatic and renal systems, as well as anti-cancer and anti-inflammatory properties [56]. Ellagic acid aglycone was the most significant contributor, supporting the observation that ellagitannin hydrolysis occurs as a response to storage conditions, potentially as a mechanism to enhance antioxidant protection or regulate oxidative stress [57]. Galloylquinic acid increased over storage, suggesting that galloyl derivatives may be actively produced or retained as part of a protective response. The volcano plot analysis further highlighted caffeic acid hexoside and flavan-3-ol derivative as significantly altered metabolites, both of which decreased over storage. The reduction in caffeic acid hexoside aligns with findings that hydroxycinnamic acids degrade due to enzymatic oxidation, contributing to reduced phenolic stability during postharvest storage [58]. According to Simões et al. [59] enzymatic activity in combination with storage temperature affects the metabolism of phenolic compounds and their concentration in leafy vegetables. Similarly, the decrease in flavan-3-ol derivative suggests a progressive depletion of flavan-3-ols, which play a key role in antioxidant defense and structural integrity [60]. These reductions indicate that phenolic metabolism in leaves undergoes active modifications over time, likely in response to oxidative stress or enzymatic breakdown processes.

### 4.3. NOX2 Inhibitory Potential of Strawberry Metabolites

According to machine learning predictions, strawberries contain a wide range of metabolites that may inhibit NOX2 activity. Despite their structural diversity, 21 of the 24 tested metabolites were characterized as active, indicating that various phytochemical classes converge on a shared ability to inhibit the NADPH oxidase (NOX2) enzyme complex and its oxidative products. These metabolites possess structural features that match known inhibitors of the NOX2 enzyme from various sources. The phenolic compounds identified in strawberries, such as caffeic acid, which is a dihydroxycinnamic acid, alongside epicatechin, which is a flavan-3-ol, demonstrate the presence of catechol moieties or polyphenolic structures. In accordance with our findings, methoxy-catechol apocynin and glycosylated polyphenol myricitrin serve as known inhibitors of NOX2-derived reactive oxygen species according to Shubina et al. [61]. The ability of these functional groups to conduct redox reactions and scavenge radicals mirrors how established NOX2 inhibitors function to inhibit the enzyme. The predicted active compounds identified by the model include flavonoids such as quercetin-3-glucuronide and kaempferol alongside tannin derivatives like procyanidin dimer, which are supported by studies showing that various polyphenols suppress NADPH oxidase activity or expression [61]. Quercetin displays inconsistent outcomes in direct NOX2 inhibition tests where full effects need metabolic activation [61]. The computational findings demonstrate that the polyphenol chemical group present in strawberries possesses essential structural features crucial for NOX2 inhibition. Strawberries are rich in antioxidants, including vitamin C, and polyphenols, along with ellagitannins [62]. The ROS-generating NOX2 enzyme system shows predicted interference abilities which parallel the antioxidant profile of strawberries. The 21 metabolites exhibit diverse characteristics, yet their molecular classifications link to specific patterns which influence the model’s predictions. Flavonoids and polyphenols like quercetin-, kaempferol- and catechin-related compounds typically display planar aromatic ring structures with numerous hydroxyl groups [63]. The molecular descriptors show low FractionCSP3 and large polar surface areas, which indicate high aromaticity and are considered essential for NOX2-inhibiting activity. The model’s main descriptors, the VSA_EState and PEOE_VSA bins, probably identified the electron-abundant aromatic areas and hydrogen-bonding abilities of these substances which support NOX2 binding or radical intermediate scavenging [14]. The phenolic acids, caffeic acids, and ellagic acids, with their glycosides, are small yet highly conjugated molecules that contain polar functional groups including carboxylic acids and multiple hydroxyl groups: these compounds show moderate molecular weight combined with high polarity and extensive negative charge distribution, which align with specific descriptor parameters (e.g., PEOE_VSA6 for near-neutral polar surfaces and MinAbsEStateIndex for strong electron-withdrawing groups) [64]. The model’s ability to predict these acids as actives demonstrates that lower-molecular-weight antioxidants meet the necessary requirements, which aligns with studies showing that phenolic acid derivatives inhibit NOX2 when hydrophobic substitutions enable membrane access [65]. The chemistries of the terpenoid-derived metabolites listed show significant differences via their largely aliphatic structure and higher FractionCSP3, which indicates more sp³ carbons, as well as an increased potential hydrophobic surface area, demonstrated by SlogP_VSA6 and other relevant descriptors. The model’s prediction of these molecules as active suggests that their lipophilic nature allows them to interact with membrane-bound oxidase complexes and inhibit NOX2. Lipophilicity is believed to improve membrane enzyme inhibitor attachment [66]. The decision boundary of the model shows different chemical signatures from the phenolic, flavonoid, and terpenoid classes (polar aromatic vs. non-polar aliphatic), leading to active predictions. Multiple chemical scaffolds can functionally reduce NOX2 activity by directly inhibiting the enzyme or acting through indirect antioxidant mechanisms [67]. The role of strawberry metabolites as antioxidants represents a common functional theme that directly relates to NOX2’s biological role. The main duty of NOX2 is to generate superoxide radicals which play a crucial part in oxidative bursts during immune defense [68]. Potent antioxidant compounds can interfere with this process by capturing produced radicals or by disrupting ROS enzyme production [69]. The active metabolites predicted in strawberries contain recognized dietary antioxidants including vitamin C (ascorbic acid), ellagic acid, catechin/epicatechin, and caffeic acid. Ascorbic acid functions by scavenging superoxide directly and recycling additional antioxidants, and its inclusion in the active list matches its role in diminishing oxidative stress within biological systems [70]. The polyphenolic compound catechins and caffeic/ferulic acid derivatives function as free radical neutralizers while also binding metal ions and controlling cell signaling pathways that respond to oxidative stress [71]. Analysis through the model identified essential molecular features such as catechol groups and conjugated double bonds, along with suitable lipophilicity, that enable molecules to effectively neutralize ROS and engage with redox enzymes [72].

The in silico results should be evaluated together with the postharvest storage modifications and biological functions of the previously mentioned metabolites. The metabolites expected to inhibit NOX2 activity include those which exhibited storage-related dynamic changes in fruits and those recognized for their plant defense functions. Data analysis revealed that caffeic acid, along with its derivative caffeic acid hexoside, faced considerable depletion throughout strawberry storage because of oxidative consumption. ML findings indicate caffeic acid as a potent NOX2 inhibitor which, when depleted during storage, reduces antioxidant capacity in fruit and diminishes the health benefits associated with NOX2 inhibition. The storage of strawberries led to a reduction in ferulic acid hexose derivative levels. Given the predicted NOX2 inhibitory activity of this compound, it can be inferred that fresh strawberries, with higher concentrations of this metabolite, may exhibit enhanced NOX2 modulation compared to those subjected to prolonged storage. During the later stages of fruit senescence, dicaffeoylquinic acid levels rise, which points toward a possible compensatory mechanism where the fruit produces alternative active antioxidants that might also inhibit NOX2. The fruit employs an inherent defense mechanism through the preservation of certain metabolites, like ellagic acid derivatives, which maintain antioxidant protection and demonstrate NOX2 inhibition capabilities when consumed. During storage, strawberry leaves contain salidroside and ellagic acid aglycone, which serve as main antioxidants for defense [73,74]. In this study, we chose to highlight only those metabolites with well-documented structures and established bioactivities in order to ensure clarity and scientific accuracy. Additional identified compounds may also hold biological relevance and merit further investigation.

## 5. Conclusions

This study provides a comprehensive evaluation of postharvest metabolic shifts in strawberry fruits and leaves using LC-MS/MS-based profiling, multivariate statistical analyses, and machine learning approaches. Significant alterations were observed in organic acids, phenolic acids, and flavonoids during storage, underscoring dynamic biochemical changes across tissues. In the fruits, the decrease in malic and caffeic acids highlighted their roles in respiration and antioxidant defense, while the accumulation of dicaffeoylquinic acid indicated a possible compensatory response to oxidative stress. In contrast, the leaves exhibited a more variable metabolic response, with fluctuations in flavonoid and phenolic acid levels, reflecting their function in storage-related stress and structural integrity. Key metabolic markers distinguishing the early and late storage stages were identified through PLS-DA and volcano plot analyses. In the fruits, metabolites such as caffeic acid, malic acid, phloridzin, and quercetin-3-glucuronide were the most indicative of storage-driven changes. Meanwhile, dynamic alterations in flavonoids and hydroxycinnamic acid derivatives reflected ongoing metabolic adjustments associated with oxidative stress and senescence. In the leaves, ellagic acid aglycone, galloylquinic acid, galloyl hexose, and flavan-3-ol derivative were identified as principal contributors to metabolic differentiation, associated with oxidative stress responses and tissue-specific defense mechanisms. Furthermore, machine learning predictions revealed that 21 out of 24 structurally diverse metabolites, including phenolic acids, flavonoids, tannins, terpenoids, organic acids, phenolic glycosides, and amino acids, were classified as potential NOX2 inhibitors. These findings suggest that strawberries are a rich source of phytochemicals capable of modulating oxidative stress through NADPH oxidase inhibition, complementing their well-established antioxidant properties. Overall, these findings emphasize the complexity of biochemical responses in both fruit and leaf tissues under storage conditions and provide valuable insights into the regulation of postharvest quality. Additionally, this integrative approach highlights the value of combining LC-MS/MS profiling and machine learning modeling to elucidate storage-related metabolic pathways and identify bioactive compounds with potential health benefits. Further biological validation is warranted to confirm the NOX2-inhibitory potential of key strawberry metabolites and explore their applications in functional foods or nutraceuticals.

To conclude, the present study not only offers insights into the temporal metabolic changes occurring in postharvest strawberry fruits and leaves but also underscores their value beyond conventional consumption. The identification of key bioactive metabolites with antioxidant properties and predicted NOX2 inhibitory activity suggests that both tissues could serve as underutilized sources of health-promoting compounds. This highlights their potential for application in the food industry as natural functional ingredients, and in the biomedical field as candidates for further exploration in anti-inflammatory or oxidative-stress-reducing interventions. The valorization of these postharvest tissues also supports sustainable and circular food system approaches, reducing waste while adding value to agricultural by-products.

## Figures and Tables

**Table 1:** Identified metabolites in strawberry fruits and leaves.

No	Tentative Identification	Group	MW	MS (*m*/*z*), ID	MS/MS	Retention Time (min)	Reference	**Strawberry Fruits** /Leaves **
1	Malic acid	Organic acids	134.0578	133.12	115.31; 89.30; 71.22	0.654	[24], https://hmdb.ca/spectra/ms_ms/2254556, accessed on 8 February 2025	+/+
2	Dehydroascorbic acid	Vitamin and vitamin derivatives	174.0165	173.01	155.22; 111.31; 87.20; 85.23	0.836	[24], https://www.hmdb.ca/spectra/ms_ms/283274, accessed on 8 February 2025	+/−
3	L-ascorbic acid	Vitamin and vitamin derivatives	176.0685	175.05	131.01; 115.20; 113.10; 87.42	1.584	[24], https://hmdb.ca/spectra/ms_ms/1473014, accessed on 8 February 2025	+/−
4	Caffeic acid	Phenolic acids	180.0423	179.04	135.11; 107.21; 91.10	11.856	[25], https://hmdb.ca/spectra/ms_ms/2234953, accessed on 8 February 2025	+/+
5	Gallic acid monohydrate	Phenolic acids	188.0321	187.16	169.24; 125.40; 97.45	4.781	[25]	+/−
6	Citric acid	Organic acids	192.0271	191.12	173.42; 129.40; 111.22	0.737	[24,26]	+/+
7	Tryptophan	Amino acids	204.0899	203.16	186.61; 142.32; 116.33	1.335	[24]	+/−
8	(-) Epicatechin	Proanthocyanidins	290.2681	289.20	245.80; 205.50; 203.21; 179.60; 109.12	1.671	[27,28]	+/+
9	Salidroside	Phenolic glycosides	300.0852	299.27	179.54; 137.00; 89.43	1.062	[12]	−/+
10	Ellagic acid aglycone	Phenolic acids	302.0067	301.31	300.51; 284.52, 257.51; 229.53; 200.34	4.101	[24]	+/+
11	Coumaric acid hexose	Phenolic acids	326.1011	325.24	163.42; 145.31	1.855	[24]	+/−
12	Galloyl hexose	Phenolic acids	332.0752	331.10	169.31; 123.50	0.837	[12]	−/+
13	Coumaroylquinic acid	Phenolic acids	338.1013	337.27	191.52; 173.50	4.100	[12]	−/+
14	Caffeic acid hexoside	Phenolic acids	342.0951	341.32	179.31; 161.30; 135.12	0.611	[29]	+/+
15	Galloylquinic acid	Phenolic acids	344.0753	343.23	191.00; 169.61; 93.42	0.800	[12]	−/+
16	p-Coumaroyl-ester	Phenolic acids	356 *	355.24	295.91; 193.41; 175.10; 134.51	3.486	[23]	−/+
17	Dihydroferulic acid 4-O-glucuronide	Hydroxycinnamic acids	372.1056	371.31	209.82; 193.51	3.564	[26]	−/+
18	Apigenin-7-O-glucoside	Flavonols	432.3775	431.28	270.82; 269.54; 225.22	2.927	[26,29]	+/−
19	Phloridzin	Flavonoid glycosides	436.1369	435.24	273.51; 167.33; 125.23	5.061	[26]	+/+
20	Ellagic acid deoxyhexoside	Hydroxycinnamic acid derivatives	448.0641	447.29	302.20; 301.50; 300.51; 257.52; 229.51	3.892	[27,30]	+/+
21	Ferulic acid hexose derivative	Hydroxycinnamic acids	450 *	449.29	355.61; 287.52; 269.52; 193.83	3.147	[22,29]	+/−
22	Kaempferol glucuronide	Flavonols	462.3604	461.25	285.50; 257.51; 229.60; 175.60; 163.51; 113.21	4.609	[24,31]	+/−
23	Quercetin hexoside	Flavonoid glycosides	464.3763	463.53	300.50; 271.61; 255.72; 179.52	4.232	[27,29,31]	+/−
24	Sesquiterpenoid	Terpenoids and related compounds	464.2628	463.55	417.81; 255.51; 161.31	5.308	[24]	+/−
25	Kaempferol hexose	Flavonols	466.1118	465.28	447.30; 285.62; 241.63; 151.40	3.333	[12,24]	−/+
26	Di-coumaroyl hexose	Hydroxycinnamic acid derivatives	472.1383	471.24	163.52; 145.31	5.236	[12]	−/+
27	Quercetin-3-glucuronide	Flavonoid glycosides	478.3598	477.27	301.63; 255.71; 179.51; 151.34; 121.30	4.253	[12,29]	+/+
29	Sapogenin	Terpenoids and related compounds	488.3515	487.54	469.81; 407.80; 135.51	6.451	[24]	+/−
30	Kaempferol acetyl glucoside	Flavonols	490.4136	489.28	447.93; 285.62; 255.42	4.957	[27]	+/−
31	Octadecatrienoic acid glycoside	Fatty acid derivatives	560.3221	559.47	513.90; 277.71; 253.71; 161.50	7.670	[12]	−/+
32	Dicaffeoylquinic acid	Phenolic acids	562.2996	561.61	515.30; 191.82; 161.42	6.961	[24]	+/−
33	Flavan-3-ol derivative	Proanthocyanidins	578.1647	577.27	425.94; 407.82; 289.60; 269.51; 147.60	3.374	[24,27]	+/+
34	Kaempferol coumaroyl hexoside	Flavonols	594.5196	593.34	447.52; 285.84; 255.41	6.126	[27]	+/+
35	Kaempferol-rutinoside	Flavonols	594.1585	593.12	547.53; 327.51; 308.80; 285.60	6.111	[32]	+/−
36	Kaempferol pentose glucuronide	Flavonols	594.1244	593.24	307.63; 285.62; 113.31	6.008	[12]	−/+
37	Q-rutinoside	Flavonoid glycosides	610.1533	609.26	301.50; 179.41; 151.42	3.856	[23]	−/+

* MW accuracy may vary for compounds with partially unknown structures or literature-based tentative identifications. ** ‘+’ indicates presence and ‘−’ indicates absence of metabolite in respective tissues (fruit or leaf).

**Table 2:** Strawberry fruit metabolites showing statistically significant changes during storage, based on one-way ANOVA followed by Tukey’s HSD post hoc test (*p <* 0.05). Among-days comparison indicates time points with significant differences in metabolite intensity.

Metabolites	*p*-Value	Significant Differences (Tukey’s HSD) *
Caffeic Acid	2.59 × 10^−7^	Day 11 vs. Day 1, Day 6 vs. Day 4, Day 11 vs. Day 4, Day 11 vs. Day 6, Day 11 vs. Day 8
Malic Acid	6.12 × 10^−5^	Day 8 vs. Day 1, Day 11 vs. Day 1, Day 8 vs. Day 4, Day 11 vs. Day 4, Day 8 vs. Day 6
Citric Acid	7.50 × 10^−5^	Day 6 vs. Day 1, Day 8 vs. Day 1, Day 6 vs. Day 4, Day 8 vs. Day 4, Day 11 vs. Day 8
Ferulic Acid Hexose Derivative	0.000481	Day 6 vs. Day 4, Day 8 vs. Day 4, Day 11 vs. Day 4
Coumaric Acid Hexose	0.003591	Day 11 vs. Day 1, Day 11 vs. Day 4
Dicaffeoylquinic Acid	0.008077	Day 6 vs. Day 1, Day 8 vs. Day 6
Kaempferol Glucuronide	0.008119	Day 4 vs. Day 1, Day 11 vs. Day 4

* Day X vs. Day Y indicates comparison of metabolite levels at day X relative to day Y.

**Table 3:** Strawberry leaf metabolites showing statistically significant changes during storage, based on one-way ANOVA followed by Tukey’s HSD post hoc test (*p <* 0.05). Among-days comparison indicates time points with significant differences in metabolite intensity.

Metabolites	*p*-Value	Significant Differences (Tukey’s HSD) *
Galloyl Hexose	5.8522 × 10^−8^	Day 4 vs. Day 1, Day 6 vs. Day 4, Day 8 vs. Day 4
Ellagic Acid Aglycone	8.7371 × 10^−8^	Day 4 vs. Day 1, Day 6 vs. Day 1, Day 8 vs. Day 1, Day 6 vs. Day 4, Day 8 vs. Day 4
Caffeic Acid Hexoside	1.0295 × 10^−5^	Day 6 vs. Day 1, Day 6 vs. Day 4, Day 8 vs. Day 6
Salidroside	2.6579 × 10^−5^	Day 4 vs. Day1, Day 6 vs. Day 1, Day 8 vs. Day 4, Day 8 vs. Day 6
Phloridzin	2.8896 × 10^−5^	Day 4 vs. Day 1, Day 6 vs. Day 4, Day 8 vs. Day 4
Malic Acid	0.00079905	Day 4 vs. Day 1, Day 6 vs. Day 4
Galloylquinic Acid	0.0035448	Day 4 vs. Day 1, Day 6 vs. Day 1
Citric Acid	0.0076111	Day 4 vs. Day 1, Day 8 vs. Day 4
Flavan-3-ol Derivative	0.018281	Day 8 vs. Day 4

* Day X vs. Day Y indicates comparison of metabolite levels at day X relative to day Y.

**Table 4:** A table of the 16 selected descriptors. The whole process was repeated 100 times, giving (4 × 100 = 400 feature-importance cycles). The ranking was performed by means of how many times each feature appeared in the feature-importance cycles.

Features’ Ranking by Importance	Feature Names (Descriptors)	Frequency of Appearance
1	BCUT2D_MWHI	381
2	PEOE_VSA6	375
3	Chi2v	357
4	BCUT2D_MRHI	298
5	qed	282
6	SMR_VSA10	278
7	VSA_EState2	272
8	SlogP_VSA6	257
9	FractionCSP3	257
10	VSA_EState6	251
11	BCUT2D_MWLOW	248
12	EState_VSA7	233
13	MinAbsEStateIndex	231
14	Chi2n	228
15	VSA_EState8	223
16	PEOE_VSA9	213

**Table 5:** A list of the strawberry-derived metabolites predicted as NOX2 inhibitors, with the corresponding SMILES annotations and ML-system predictions. The @ and @@ symbols in the SMILES strings indicate stereochemistry and are part of the standard notation used in cheminformatics to describe chiral centers.

Compound	Chemical Formula	SMILES	ML-System Prediction
Epicatechin	C15H14O6	O[C@H]1CC2=C(O)C=C(O)C=C2O[C@H]1C1=CC(O)=C(O)C=C1	Active
Apigenin-7-O-glucoside	C21H20O10	OCC1OC(OC2=CC(O)=C3C(=O)C=C(OC3=C2)C2=CC=C(O)C=C2)C(O)C(O)C1O	Active
Sesquiterpenoid	C15H18O3	C[C@@H]1[C@@H]2CC[C@]3(C)C=CC(=O)C(C)=C3[C@@H]2OC1=O	Not Active
Sapogenin	C30H50O3	C[C@H]1CC[C@@]2([C@H]([C@H]3[C@@H](O2)C[C@@H]4[C@@]3(CC[C@H]5[C@H]4CCC6[C@@]5(CCCC6)C)C)C)OC1	Active
Salidroside	C14H20O7	OCC1OC(OCCC2=CC=C(O)C=C2)C(O)C(O)C1O	Active
Quercetin-3-glucuronide	C21H18O13	OC1C(O)C(OC2=C(OC3=CC(O)=CC(O)=C3C2=O)C2=CC=C(O)C(O)=C2)OC(C1O)C(O)=O	Active
Procyanidin dimer	C30H26O12	[H][C@]1([C@@H](O)[C@H](OC2=CC(O)=CC(O)=C12)C1=CC(O)=C(O)C=C1)C1=C2O[C@@H]([C@H](O)[C@@]([H])(C3=C(O)C=C(O)C4=C3O[C@@H]([C@H](O)C4)C3=CC(O)=C(O)C=C3)C2=C(O)C=C1O)C1=CC(O)=C(O)C=C1	Active
p-Coumaroyl-ester	C16H14O5	O[C@@H]1C[C@](O)(C[C@@H](OC(=O)\C=C/C2=CC=C(O)C(O)=C2)[C@@H]1O)C(O)=O	Active
Malic acid	C4H6O5	OC(CC(O)=O)C(O)=O	Not Active
L-ascorbic acid	C6H8O6	OCC(O)C1OC(=O)C(O)=C1O	Active
Kaempferol	C15H12O6	OC1=CC=C(C=C1)C1=C(O)C(=O)C2=C(O1)C=C(O)C=C2O	Active
Kaempferol acetylhexose	C23H22O12	OCC1OC(OC2C(O)C(O)C(CO)OC2OC2=C(OC3=CC(OC4OC(C(O)C(O)C4O)C(O)=O)=CC(O)=C3C2=O)C2=CC=C(O)C=C2)C(O)C(O)C1O	Active
Isorhamnetin hexose	C22H20O12	COC1=C(OC2OC(CO)C(O)C(O)C2O)C=CC(=C1)C1=C(O)C(=O)C2=C(O)C=C(O)C=C2O1	Active
Galloylquinic acid	C16H12O10	OS(=O)(=O)OC(=O)C1=CC=CC=C1	Active
Gallic acid monohydrate	C7H6O5	C(O)(=O)C1=CC(O)=C(O)C(O)=C1.[H]O[H]	Active
Ellagic acid deoxyhexose	C20H16O12	OC1=CC=C(\C=C\C2=CC(O)=CC(O)=C2)C=C1	Active
Ellagic acid	C14H6O8	OC1=C(O)C2=C3C(=C1)C(=O)OC1=C3C(=CC(O)=C1O)C(=O)O2	Active
Dicaffeoylquinic acid	C25H24O12	O[C@H]1[C@H](OC(=O)\C=C\C2=CC=C(O)C(O)=C2)C[C@@](O)(C[C@H]1OC(=O)\C=C\C1=CC=C(O)C(O)=C1)C(=O)O	Active
Dehydroascorbic acid	C6H6O6	[H][C@@]1(OC(=O)C(=O)C1=O)[C@@H](O)CO	Not Active
Coumaroylquinic acid	C16H16O9	O[C@@H]1C[C@@](O)(C[C@@H](OC(=O)\C=C\C2=CC=C(O)C=C2)[C@H]1O)C(O)=O	Active
Citric acid	C6H8O7	OC(=O)CC(O)(CC(O)=O)C(O)=O	Not Active
Catechin	C15H14O6	O[C@H]1CC2=C(O)C=C(O)C=C2O[C@@H]1C1=CC(O)=C(O)C=C1	Active
Caffeic acid	C9H8O4	OC(=O)\C=C\C1=CC(O)=C(O)C=C1	Active
Tryptophan	C11H12N2O2	NC(CC1=CNC2=C1C=CC=C2)C(O)=O	Active

## Data Availability

All the data presented in this study are available within the article.

## Supplementary Materials

The following supporting information can be downloaded at: https://www.mdpi.com/article/10.3390/metabo15050321/s1, Figure S1: Box plots showing temporal variation in key metabolites in strawberry fruit during storage: (a) caffeic acid, (b) malic acid, (c) citric acid, (d) coumaric acid hexose, (e) ferulic acid hexose derivative, (f) kaempferol glucuronide, (g) dicaffeoylquinic acid; Figure S2: Permutation test and model validation parameters (R^2^, Q^2^, and classification accuracy) for PLS-DA model applied to strawberry fruit metabolite data during storage; Figure S3: Box plots showing temporal variation in key metabolites in strawberry leaves during storage: (a) malic acid, (b) citric acid, (c) salidroside, (d) ellagic acid aglycone, (e) galloylquinic acid, (f) galloyl hexose, (g) caffeic acid hexoside, (h) flavan-3-ol derivative, (i) phloridzin; Figure S4: Permutation test and model validation parameters (R^2^, Q^2^, and classification accuracy) for PLS-DA model applied to strawberry leaf metabolite data during storage; Figure S5: Variation in descriptors among studied categories (active/not active); Table S1: Differentially altered metabolites in strawberry fruit during storage (day 1 vs. day 11) based on volcano plot analysis, including log2 fold change and statistical significance (*p*-value); Table S2: Differentially altered metabolites in strawberry leaves during storage (day 1 vs. day 8) based on volcano plot analysis, including log2 fold change and statistical significance (*p*-value).
