# Supplementary material for: Exploring Postharvest Metabolic Shifts and NOX2 Inhibitory Potential in Strawberry Fruits and Leaves via Untargeted LC-MS/MS and Chemometric Analysis
Source: Metabolites. 2025 May 13;15(5):321. doi: 10.3390/metabo15050321 (PMC12114147; doi:10.3390/metabo15050321)
Supplement: Supplementary file 1 [file metabolites-15-00321-s001.zip › metabolites-3597995-supplementary.pdf]

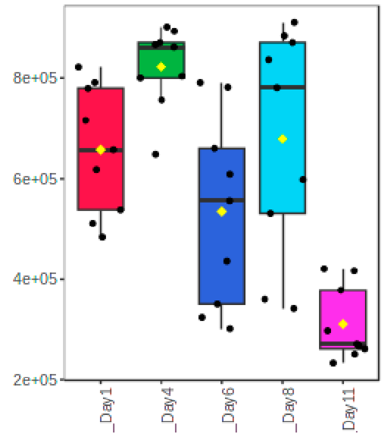
8e+05
6e+05
4e+05
2e+05
_Day1
_Day4
_Day6
_Day8
_Day11
(a)

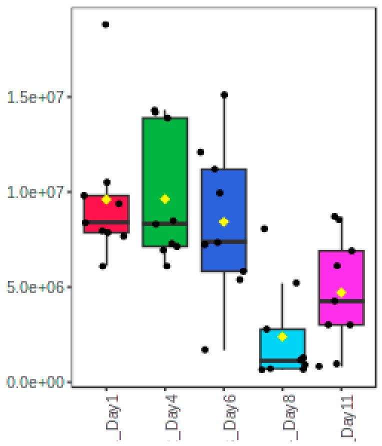
1.5e+07
1.0e+07
5.0e+06
0.0e+00
_Day1
_Day4
_Day6
_Day8
_Day11
(b)

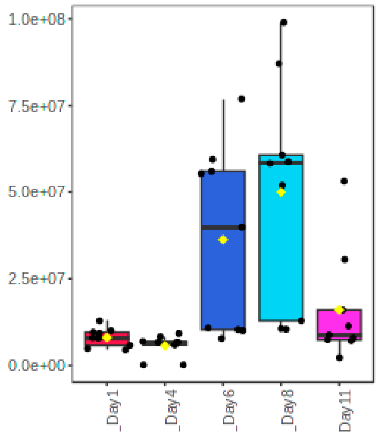
1.0e+08
7.5e+07
5.0e+07
2.5e+07
0.0e+00
_Day1
_Day4
_Day6
_Day8
_Day11
(c)

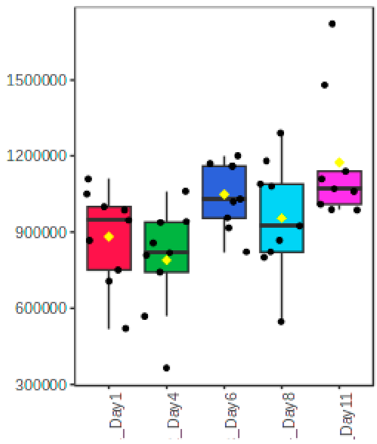
1500000
1200000
900000
600000
300000
_Day1
_Day4
_Day6
_Day8
_Day11
(d)

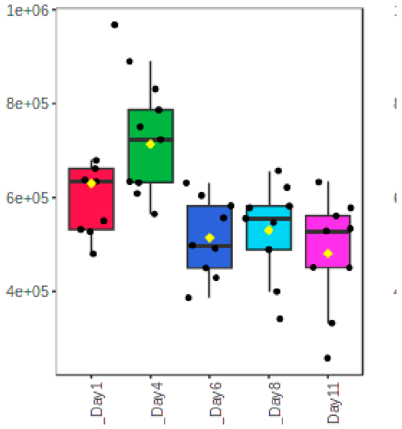
1e+06
8e+05
6e+05
4e+05
_Day1
_Day4
_Day6
_Day8
_Day11
(e)

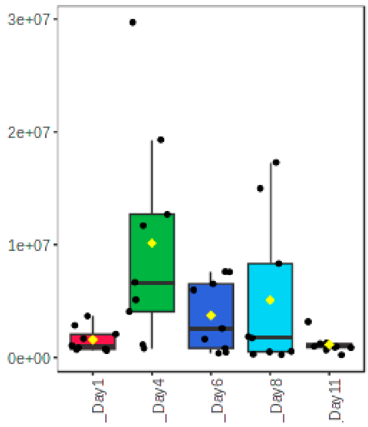
3e+07
2e+07
1e+07
0e+00
_Day1
_Day4
_Day6
_Day8
_Day11
(f)

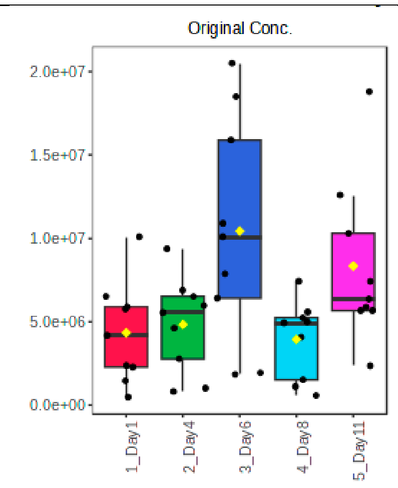

(g)

Figure S1. Box plots showing the temporal variation of key metabolites in strawberry fruit during storage: (a) caffeic acid, (b) malic acid, (c) citric acid, (d) coumaric acid hexose, (e) ferulic acid hexose derivative, (f) kaempferol glucuronide, and (g) dicaffeoylquinic acid.

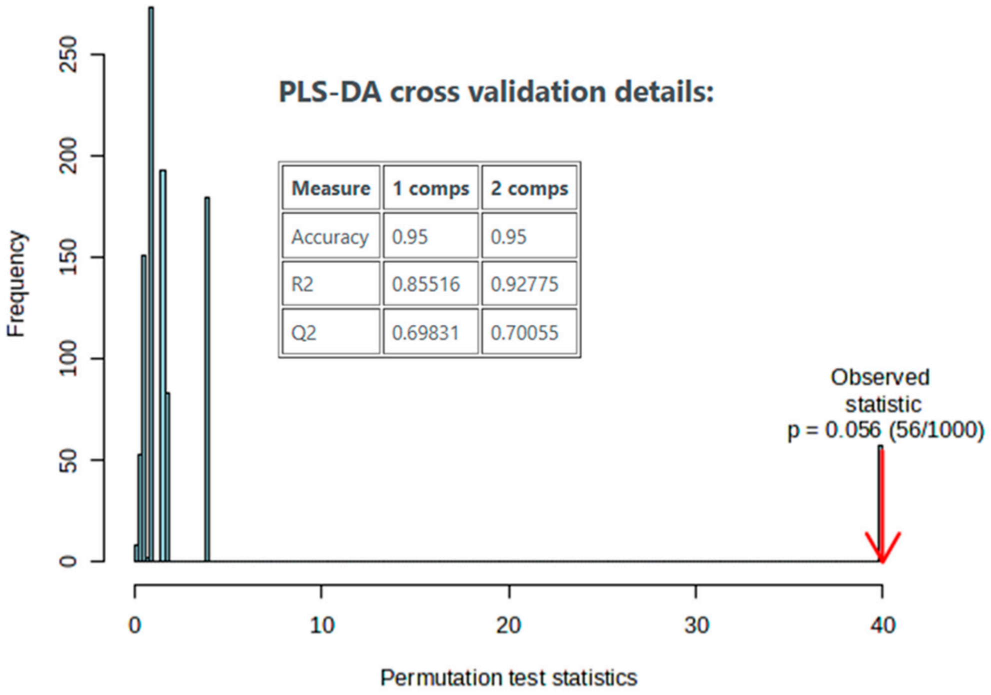

Figure S2. Permutation test and model validation parameters (R^2, Q^2, and classification accuracy) for the PLS-DA model applied to strawberry fruit metabolite data during storage.

Table S1. Differentially altered metabolites in strawberry fruit during storage (Day 1 vs. Day 11) based on volcano plot analysis, including log2 fold change and statistical significance (p-value).

Metabolite	log2(FC)	p-value
Caffeic acid	1.0807	4.1135e-05
Quercetin-3-Glucuronide	-1.9974	0.00028795
Phloridzin	-0.75962	0.0027561
Coumaric Acid Hexose	-0.4124	0.010365
Malic Acid	1.0272	0.010613
Caffeic acid hexoside	-0.80234	0.039984
Flavan-3-ol Derivative	-0.43944	0.042048
Ferulic Acid Hexose Derivative	0.3899	0.042154

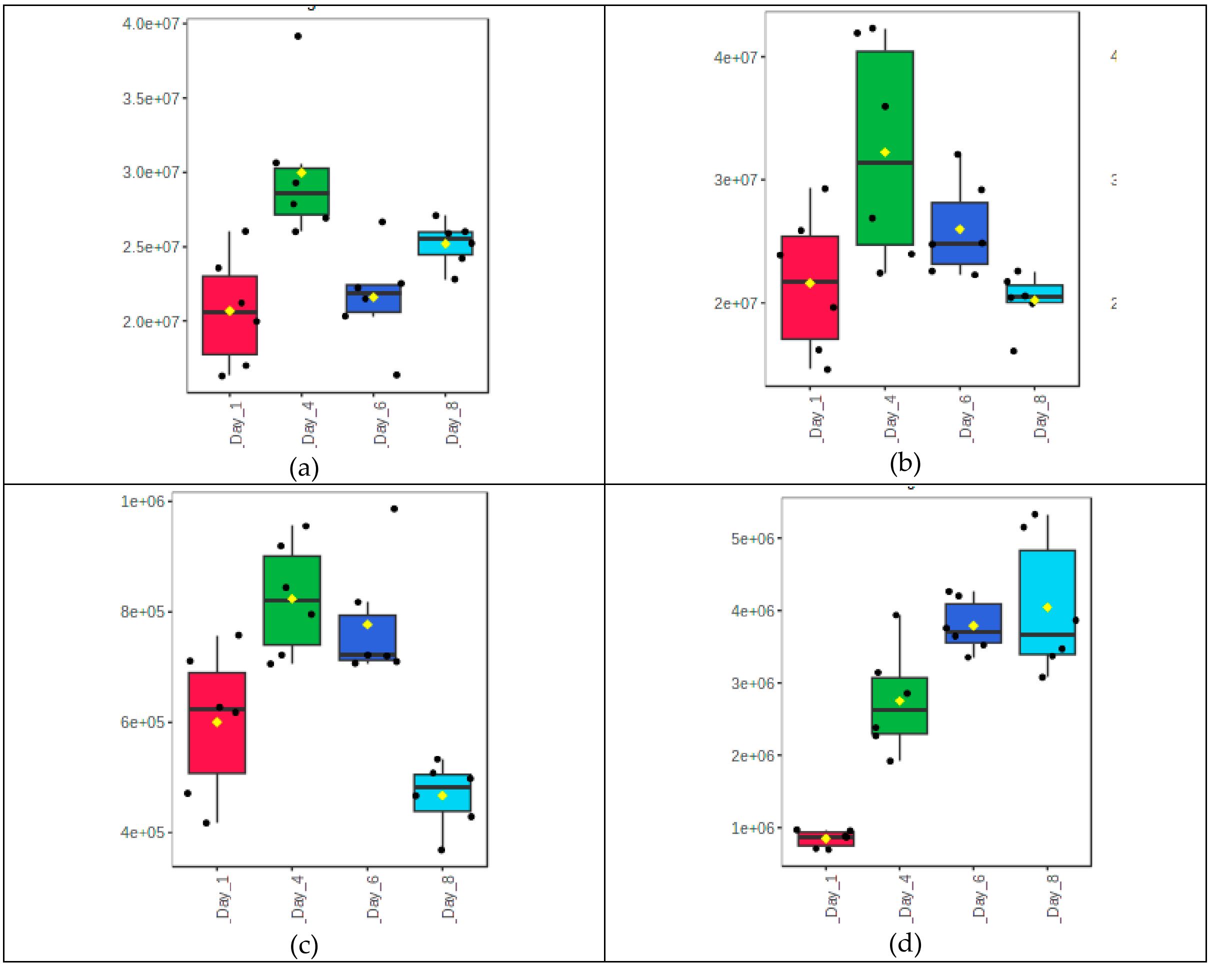

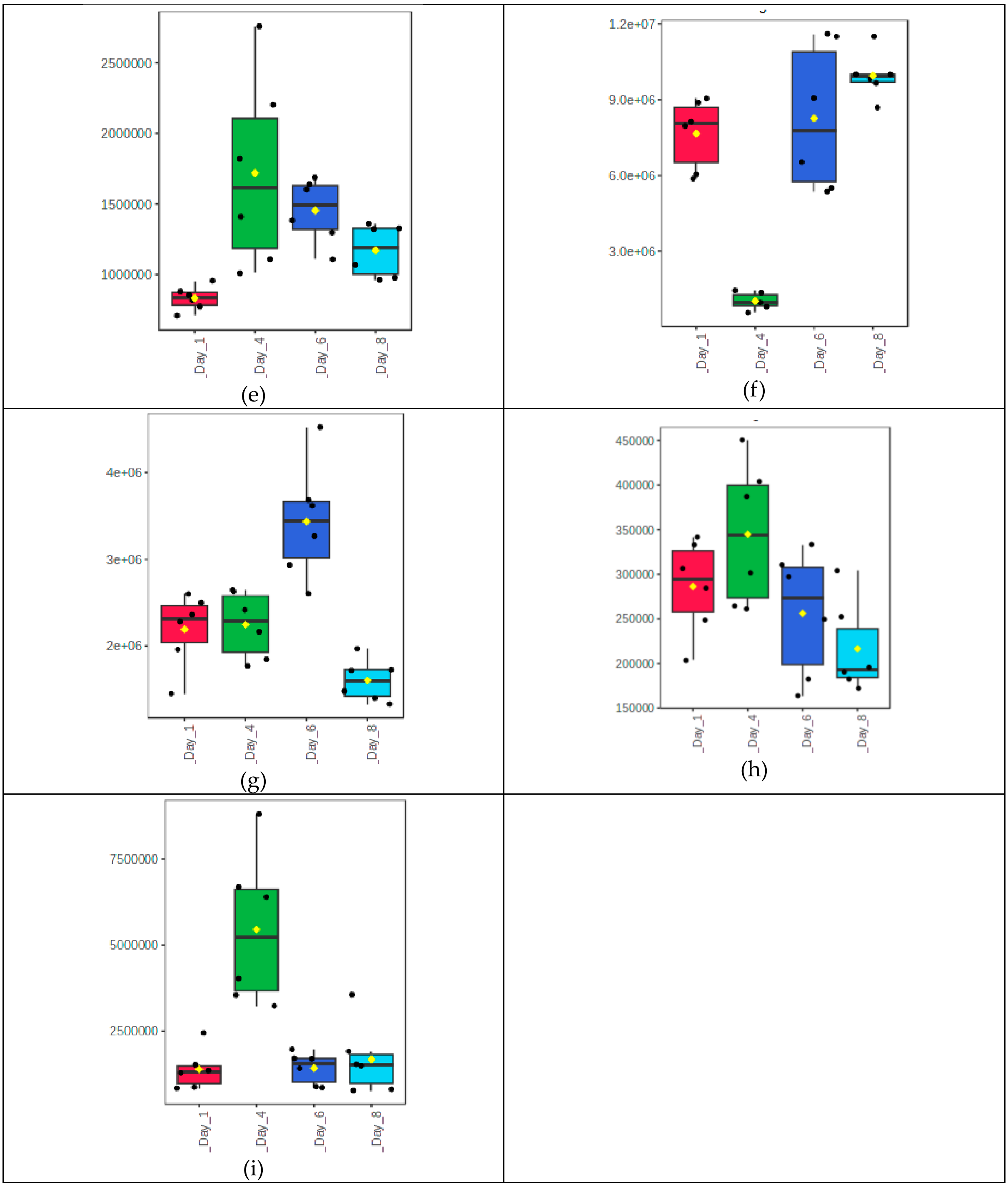

Figure S3. Box plots showing the temporal variation of key metabolites in strawberry leaves during storage: (a) malic acid, (b) citric acid, (c) salidroside, (d) ellagic acid aglycone, (e) galloylquinic acid, (f) galloyl hexose, (g) caffeic acid hexoside, (h) flavan-3-ol derivative, and (i) phloridzin.

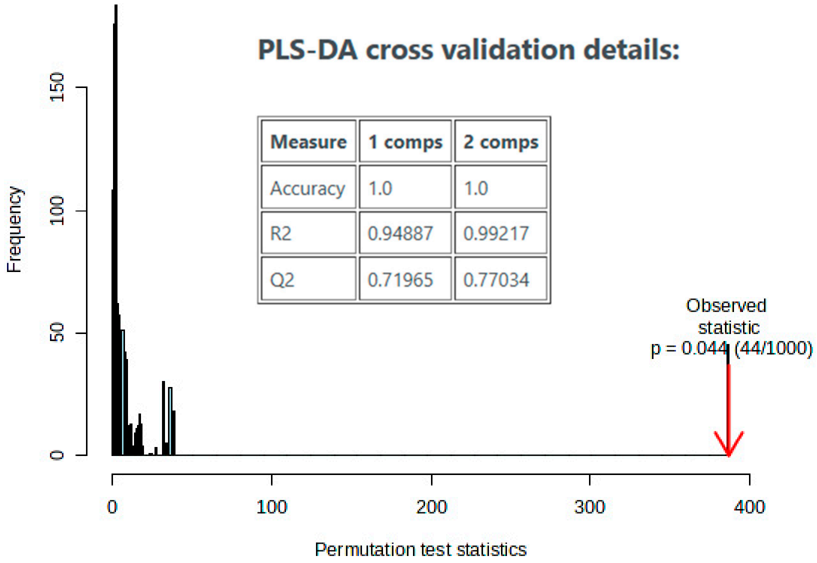

Figure S4. Permutation test and model validation parameters (R^2, Q^2, and classification accuracy) for the PLS-DA model applied to strawberry leaf metabolite data during storage.

Table S2. Differentially altered metabolites in strawberry leaves during storage (Day 1 vs. Day 8) based on volcano plot analysis, including log2 fold change and statistical significance (p-value).

Metabolite	log2(FC)	p-value
ellagic acid aglycone	-2.2531	0.002165
Galloylquinic acid	-0.49152	0.002165
Octadecatrienoic acid glycoside	0.70969	0.008658
Galloyl hexose	-0.37733	0.012907
Caffeic acid hexoside	0.44946	0.041126
flavan-3-ol derivative	0.40333	0.041126

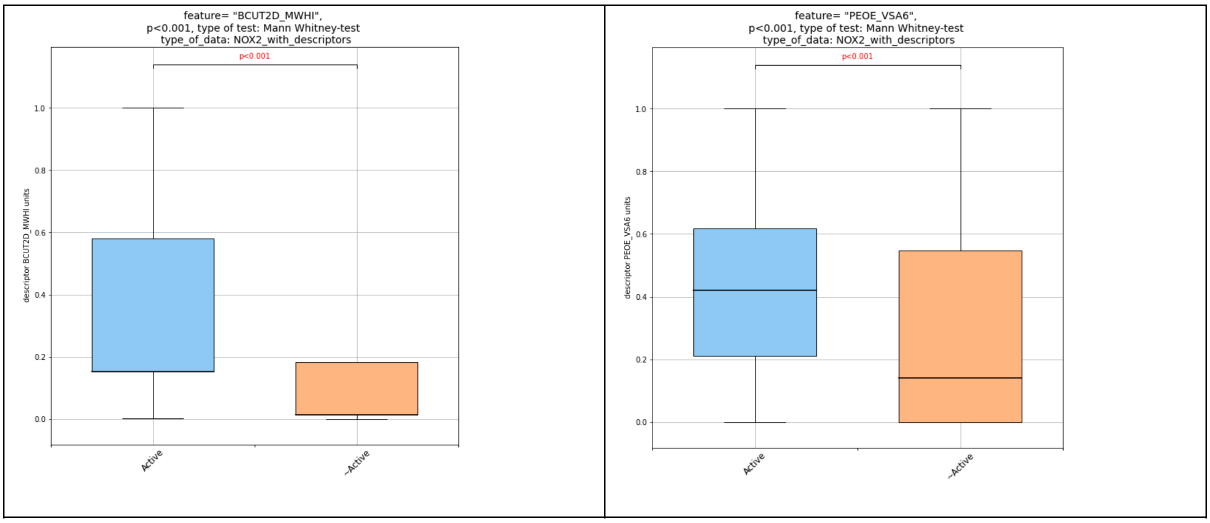

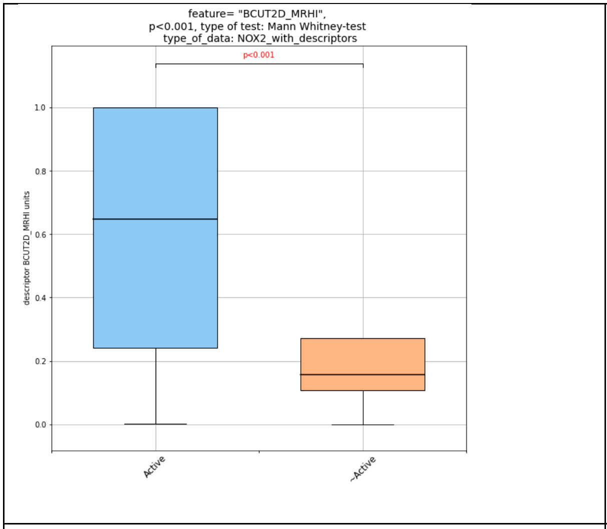
feature= "BCUT2D_MRHI",
p<0.001, type of test: Mann Whitney-test
type_of_data: NOX2_with_descriptors
p<0.001
descriptor BCUT2D_MRHI units
0.0
0.2
0.4
0.6
0.8
1.0
Active
~Active

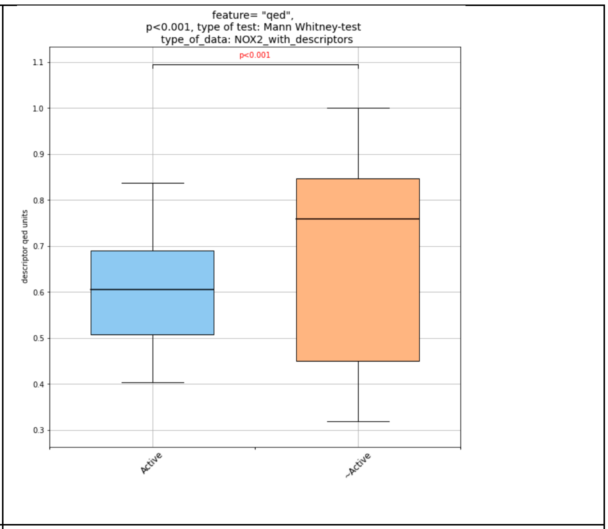
feature= "qed",
p<0.001, type of test: Mann Whitney-test
type_of_data: NOX2_with_descriptors
p<0.001
descriptor qed units
0.3
0.4
0.5
0.6
0.7
0.8
0.9
1.0
1.1
Active
~Active

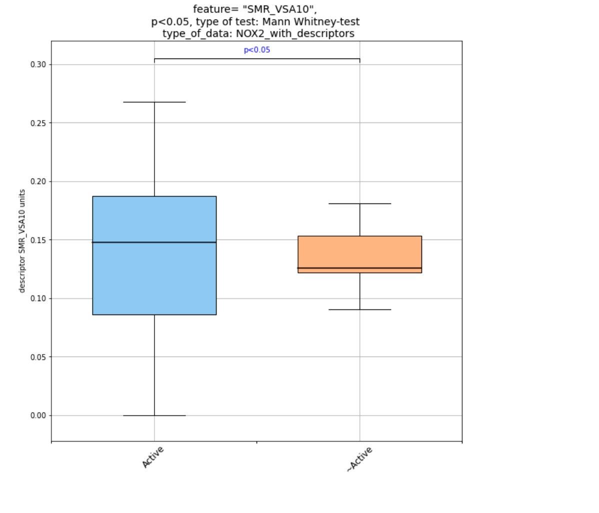
feature= "SMR_VSA10",
p<0.05, type of test: Mann Whitney-test
type_of_data: NOX2_with_descriptors
p<0.05
descriptor SMR_VSA10 units
0.00
0.05
0.10
0.15
0.20
0.25
0.30
Active
~Active

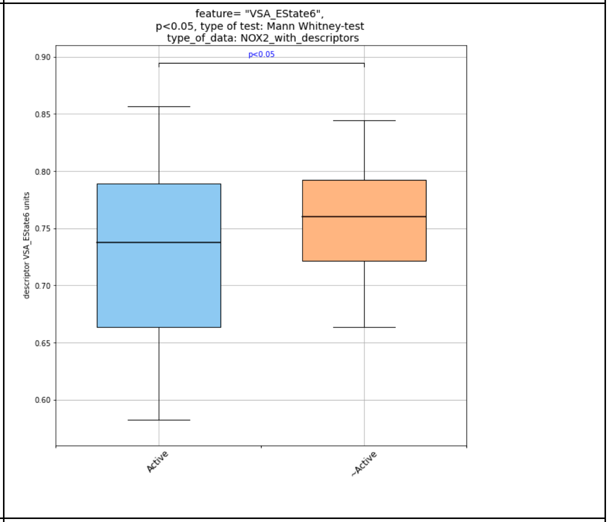
feature= "VSA_EState6",
p<0.05, type of test: Mann Whitney-test
type_of_data: NOX2_with_descriptors
p<0.05
descriptor VSA_EState6 units
0.60
0.65
0.70
0.75
0.80
0.85
0.90
Active
~Active

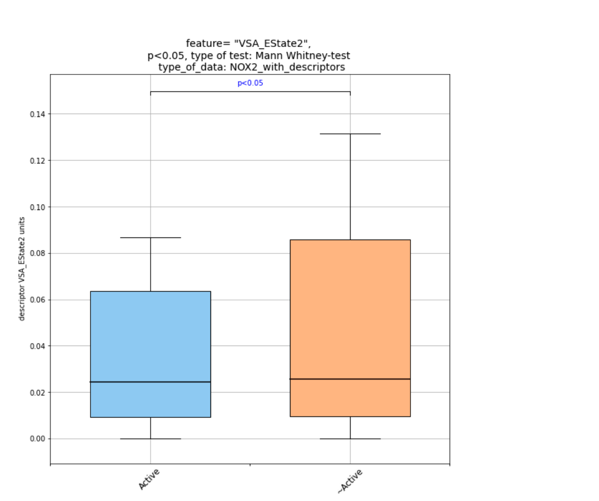
feature= "VSA_EState2",
p<0.05, type of test: Mann Whitney-test
type_of_data: NOX2_with_descriptors
p<0.05
descriptor VSA_EState2 units
0.00
0.02
0.04
0.06
0.08
0.10
0.12
0.14
Active
~Active

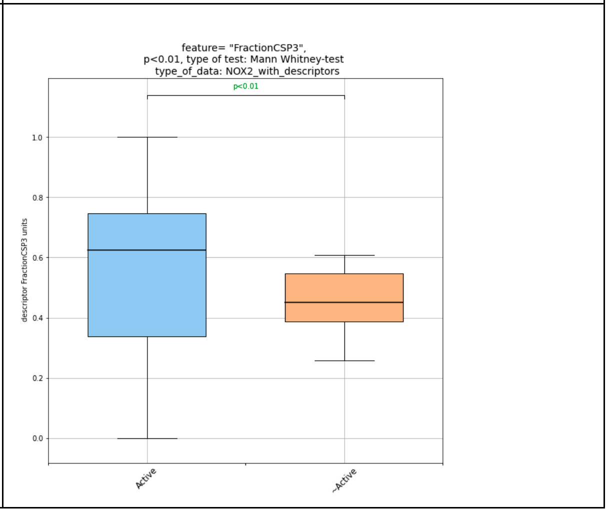
feature= "FractionCSP3",
p<0.01, type of test: Mann Whitney-test
type_of_data: NOX2_with_descriptors
p<0.01
descriptor FractionCSP3 units
0.0
0.2
0.4
0.6
0.8
1.0
Active
~Active

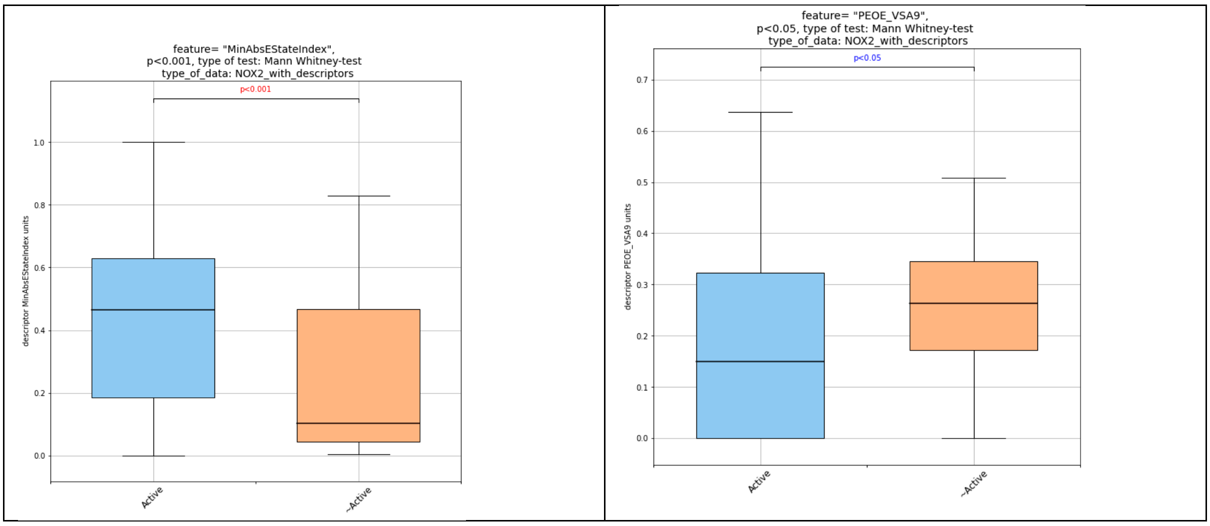

Figure S5. Variation of descriptors among the studied categories (Active/Not active).
